# Spatial clustering of esophageal cancer in central Oromia, Ethiopia: A spatial autocorrelation and scan statistics analysis

**DOI:** 10.1371/journal.pone.0357042

**Published:** 2026-09-15

**Authors:** Teresa Kisi Beyen, Edom Seife, Biniyam Tefera Deressa, Girma Taye, Adamu Addissie

**Affiliations:** 1 Department of Public Health, College of Health Science, Arsi University, Asella, Ethiopia; 2 School of Public Health, College of Health Science, Addis Ababa University, Addis Ababa, Ethiopia; 3 Global & Planetary Health Working Group, Institute of Medical Epidemiology, Biostatistics and Informatics, Martin Luther University Halle-Wittenberg, Halle (Saale), Germany; 4 College of Health Science, Addis Ababa University, Addis Ababa, Ethiopia; 5 Adama Hospital Medical College, Adama, Ethiopia; 6 Department of Preventive Medicine, School of Public Health, College of Health Science, Addis Ababa University, Addis Ababa, Ethiopia; SPHMMC: St Paul’s Hospital Millennium Medical College, ETHIOPIA

## Abstract

**Introduction:**

In Ethiopia, according to the GLOBOCAN 2022 report, there were 1,494 cases of esophageal cancer and 1,422 deaths attributed to the disease. The incidence and mortality of esophageal cancer are projected to increase to 3,210 and 3,057 cases by 2040, respectively. Approximately 68.3% of the cases were reported from the Arsi and Bale Zones, which also had the highest disease burden and mortality. Thus, undertaking a thorough study to identify hotspot areas for local preventative and control actions is important.

**Objective:**

To assess the spatial clustering of esophageal cancer in Central Oromia, Ethiopia.

**Methods:**

A cross-sectional study design was employed to investigate the spatial clustering of esophageal cancer patients. Data were collected from records of 367 patients who visited four hospitals (namely, Tikur Anbessa Specialized Hospital, Adama Hospital Medical College, Asella Teaching and Referral Hospital, and Meda Walabu University General Hospital) between April 3, 2019, and August 25, 2025. The kebele, woreda, and zone of each patient were collected from the records and linked to Ethiopian shapfiles obtained from the Ethiopian Space Science and Geospatial Institute (SSGI). The spatial inequality of esophageal cancer was measured using the Gini coefficient; while spatial autocorrelation was assessed using Moran’s I. The risks within clusters were quantified using relative risk (RR) with its 95% confidence interval, as well as log-likelihood ratio (LLR) and p-values.

**Results:**

The annual incidence of esophageal cancer in the study area was 2.60 cases per 100,000 populations [95% CI: 2.34, 2.88]. The Gini coefficient revealed spatial inequality in the distribution of cases at both the district (G = 0.33; 95% CI: 0.24, 0.40) and kebele levels (G = 0.38; 95% CI: 0.33, 0.43). The Moran’s I showd significant positive spatial autocorrelation at the kebele level (I = 0.17, p-value = 0.037). The risk of developing esophageal cancer was high in Robe district [RR = 1.93; 95% CI: 1.43, 2.61; LLR = 6.82; p-value = 0.022], Robe 01 kebele [RR = 7.8; 95% CI: 4.57, 13.31; LLR = 16.35; p-value = 0.000024], and Kula 01 kebele [RR = 9.96; 95% CI: 4.45, 22.32; LLR = 8.35; p-value = 0.034]. In contrast, 17 districts [RR = 0.55; 95% CI: 0.41, 0.73; LLR = 9.31; p-value = 0.0051] and 52 kebeles [RR = 0.55; 95% CI: 0.41, 0.73; LLR = 0.87; p-value = 0.014] were identified as low-risk areas.

**Conclusion and recommendation:**

Esophageal cancer showed uneven spatial distribution and significant autocorrelation in the study area. The presence of both high- and low-risk areas indicated significant geographic disparities. Robe district had nearly twice the risk, Robe 01 kebele had more than seven times the risk, and Kula 01 kebele had nearly ten times the risk of developing esophageal cancer compared to other districts and kebeles, respectively. Allocating diagnostic resources to health facilities serving high-risk districts and kebeles, promoting community health education on modifiable risk factors, and investigating individual-level risk factors are required to explain the observed clustering patterns.

## Introduction

Globally, the burden of cancer has increased substantially over recent decades, reaching an estimated 19,976,499 new cases and 9,743,832 deaths in 2022 [1], compared with 18.1 million new cases and 9.6 million deaths in 2018 [1–5]. If current trends persist, the global cancer burden is projected to more than double by 2050 [1,4].

In response to the growing burden of cancer and other non-communicable diseases, Sustainable Development Goal (SDG) target 3.4 aims to reduce premature mortality from NCDs by 30% by 2030 while promoting mental health and overall well-being worldwide [6].

Esophageal cancer is a malignant tumor, mainly squamous cell carcinoma (SCC) or adenocarcinoma (AC), arising from the esophageal lining. Like other cancers, it involves uncontrolled cell growth and can be fatal if not managed [7–10]. Although its global ranking has varied over the years, esophageal cancer remained the eleventh most common cause of morbidity and the seventh leading cause of cancer-related mortality worldwide in 2022 [1,4,11–14].

In 2022, an estimated 510,727 new cases (2.6%) of esophageal cancer and 445,109 deaths (4.6%) were reported worldwide [1]. The highest incidence rates occurred in Eastern Asia (248,384 cases), South-Central Asia (114,956 cases), South America (21,888 cases), Eastern Africa (17,936 cases), and Southern Africa (3,599 cases). The age-standardized incidence rate ranged from 4.5 per 100,000 in Sub-Saharan Africa to 11 per 100,000 in East Asia [15]. The disease was particularly prevalent in Central Asia (22.51%), known as the esophageal cancer belt, and in Eastern Africa’s esophageal cancer corridor (3%) [16]. Recent evidence showed that the area from Ethiopia down to South Africa is still experiencing unusually high rates of esophageal squamous cell carcinoma (ESCC) [17].

In Ethiopia, esophageal cancer ranked fourteenth in incidence, with 1,494 new cases (2.3%), and tenth in new mortality, with 1,422 deaths (2.2%), in 2022 resulting in five year prevalence of 1.5 per 100,000 (2,427) [1,11,18]. By 2040, the numbers are projected to rise to approximately 3,210 new cases and 3,057 deaths, respectively [18].

Studies have shown that the number of esophageal cancer cases is increasing in the country. More than three-fourth (75.9%) of the patients were reported from the two regions (i.e., Oromia and Somali) [19]. About 30.26% to 68.3% of esophageal cancer cases were reported from Oromia Regional State [18,20], while 26% of patients were from Somali Regional State (26%) [19]. Moreover, the largest proportion of patients (68.3%) within Oromia Regional State was reported from two zones, Arsi and Bale [20] where the study was designed.

Different studies revealed that esophageal cancer distributions vary based on geographical locations [15,19–26]. The research conducted in Canada showed the spatial clustering of esophageal cancer patients [27]. Ethiopia (Arsi/Bale regions), Kenya (Eldoret, Tenwek), Malawi, Uganda, Mozambique, Zimbabwe and Tanzania (Kilimanjaro) form part of East Africa’s high-risk corridor (with more than 82% of case in East Africa) for esophageal squamous cell carcinoma (ESCC) while Central Asia created an esophageal cancer belt [16,28,29].

In Ethiopia, only a few studies have reported regional and zonal variations in the distribution of esophageal cancer cases. However, no study to date has employed statistical modeling to investigate the spatial clustering of the disease. Instead, previous studies have mainly described spatial variation using proportional distributions across regions [30–38] and zones [30,32,35,39–42].

Despite the rising incidence of esophageal cancer in Ethiopia and high burden in Oromia Regional State, particularly in the study area, limited attention and resources have been devoted to identifying its geographical clusters.

In Ethiopia, little is known regarding the spatial distribution and clustering of esophageal cancer, particularly within the study area. Thus, research that incorporates local contexts and employs spatial modeling to detect areas of elevated risk is essential for designing effective, locally tailored prevention and control strategies for esophageal cancer. Accordingly, this study was designed to identify hotspots, cold spots, outliers, and the overall geospatial variation of esophageal cancer, addressing the limited research that has focused on its spatial clustering in Ethiopia.

The findings of this study can serve as valuable input for policymakers and planners from both governmental and non-governmental sectors, as well as for future research. Moreover, the results will assist program managers and policymakers at various levels of the health system in designing evidence-based interventions that are responsive to the local context.

## Research questions and hypotheses

### Research questions

- Is esophageal cancer spatially clustered within the study area?
- Is there an elevated risk of esophageal cancer in the identified spatial clusters?

### Hypotheses

**H**_**o1**_: There is no spatial clustering of esophageal cancer in the study area.

**H**_**A1**_: There is significant spatial clustering of esophageal cancer in the study area.

**H**_**o2**_: The risk of esophageal cancer inside the scanning window is equal to the risk outside.

**H**_**A2**_: The risk of esophageal cancer inside the scanning window is significantly higher (or lower) than the risk outside.

## Materials and methods

### Study area

This study was conducted within five adjoining zones in central Oromia, Ethiopia, namely Arsi, West Arsi, Bale, East Bale, and East Shewa.

The Arsi Zone, located in central Oromia, is bordered by Bale to the south, West Arsi to the southwest, East Shewa to the northwest, and West Hararghe to the north and east. It spans 19,825.22 km² and is divided into 27 districts and three urban administrations, containing 573 Kebeles. With an estimated population of 4,308,455 at the start of 2025, its administrative capital is Asela Town, which had an estimated 110,161 residents. The healthcare infrastructure includes nine hospitals, 112 health centers, and 540 health posts.

The West Arsi Zone is located in the Oromia Region of Ethiopia. Covering 11,776.72 km², it contains 12 districts (woredas). Its population was estimated to be over 3.6 million at the start of 2025. The administrative capital, Shashamane, had a projected population of 186,214. The zone’s healthcare system comprises one referral hospital, three general hospitals, four primary hospitals, 97 health centers, and 321 health posts.

Bale is the second-largest zone in the Oromia Regional State of Ethiopia, with a total area of 63,555 km². Its borders are defined by natural features and adjacent Zones: the East Borena to the south, the West Arsi Zone to the west, the Arsi Zone to the north, the West and East Hararghe Zones to the northeast, and the Somali Region to the east. The zone’s administrative structure includes 18 districts and two urban administrative centers, encompassing 351 rural and 20 urban kebeles. A public health system, which serves an estimated population of 1.5 million, includes six hospitals (three general and three primary), 55 health centers, and 205 health posts.

The East Bale Zone is one of the administrative zones in Ethiopia’s Oromia Region. It was established from the core Bale Zone to facilitate more localized governance. Its capital is Ginir, a town known for its strategic importance. As the name suggests, the zone occupies the eastern portion of the former, larger Bale territory. It is bordered by the Arsi Zone to the north, the original Bale Zone to the west, and the Somali Region to the south and east. The zone comprises eight weredas (districts) and a healthcare system that includes one general hospital, one primary hospital, 32 health centers, and 171 health posts, serving a population of over 750 thousand people.

**Fig 1:**
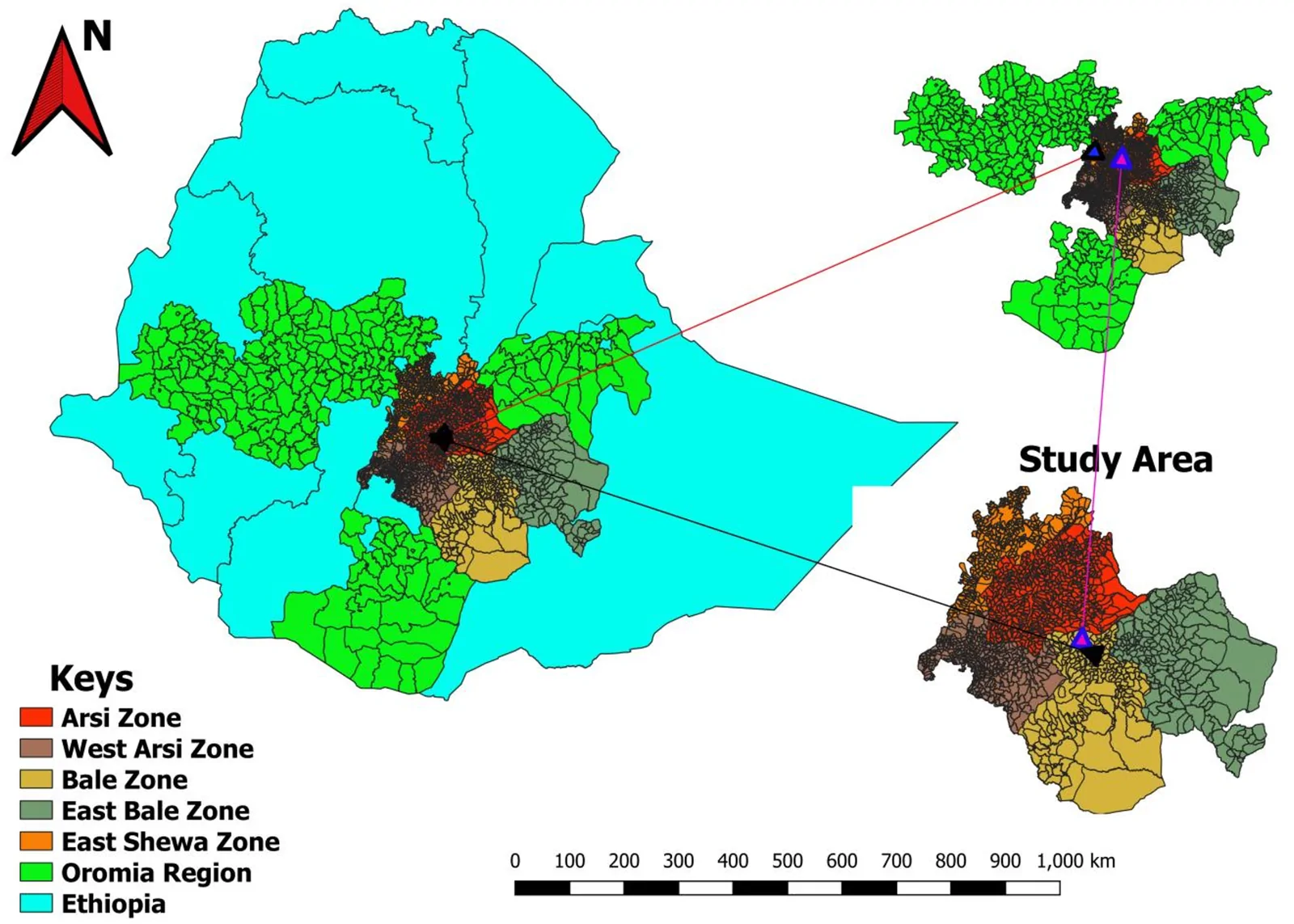
Showing the location and boundaries of study area, 2025.

The East Shewa Zone is located in central Oromia. It is bordered by the West Arsi Zone to the south, the Southern Nations, Nationalities, and Peoples’ Region to the southwest, the West Shewa Zone and the Oromia Special Zone Surrounding Finfinne to the west, the North Shewa Zone to the northwest, the Amhara Region to the north, and the Afar Region to the northeast. Its capital is Adama. The zone comprises 26 woredas (districts) and a healthcare infrastructure of eight hospitals, 84 health centers, and 330 health posts, serving a population of over two million [43] (Fig 1).

**Fig 2:**
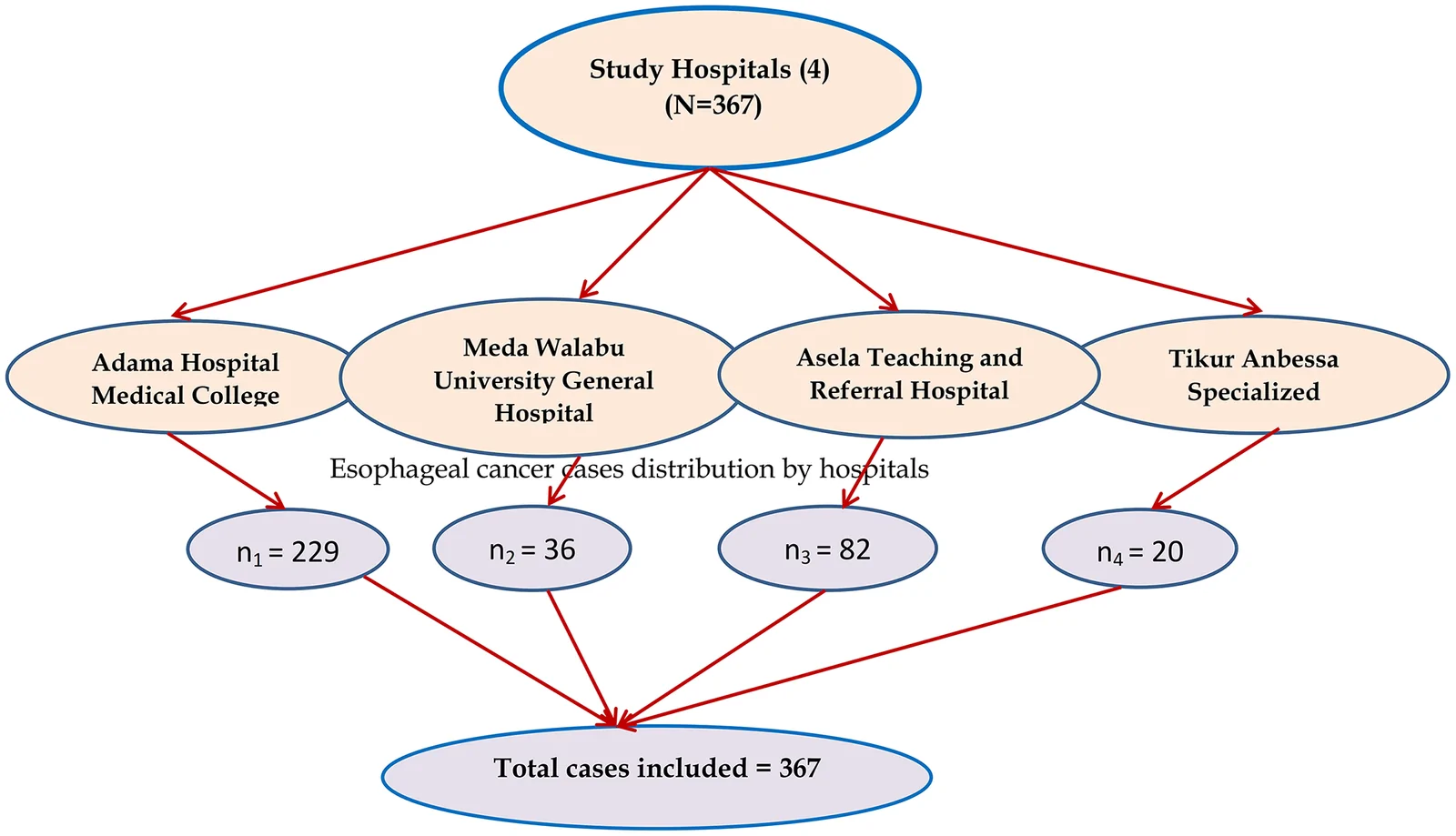
Schematic presentation of the number esophageal cancer cases included in the study by hospital, 2025 (n = 367).

For this study, three hospitals in the area, Adama Hospital Medical College, Asella Teaching and Referral Hospital, and Meda Walabu University General Hospital, were identified as providers of diagnosis and treatment for esophageal cancer, referring patients to Tikur Anbessa Specialized Hospital in Addis Ababa (Fig 2).

### Study design and period

A cross-sectional study design was employed to determine the spatial clustering of esophageal cancer. This study identified high-risk clusters using secondary data collected from patients who visited the four hospitals. However, the study design was insufficient to establish the causal pathways underlying the clusters of esophageal cancer case. Data collection took place from December 15, 2024, to August 25, 2025.

### Source population and eligibility

The source population consisted of all adult residents (≥18 years) with esophageal cancer from the five zones (Arsi, West Arsi, Bale, East Bale, and East Shewa) who visited the selected public hospitals. Eligible participants were adults (≥18 years) from these zones with an endoscopic and/or histological confirmation of esophageal cancer who attended the study hospitals between April 3, 2019, and August 25, 2025. Patients were excluded if their medical records were incomplete, specifically if they lacked diagnosis information, a known residence (Kebele), or contact details. Duplicate records were identified and excluded by patients’ card numbers.

### Sample size determination and sampling techniques

The sample size was estimated using the StatsToDo online software, which calculates sample size for Poisson-distributed count data [44]. The sample size calculation procedure followed the assumptions of the Poisson distribution, as esophageal cancer is a rare disease. The calculation was based on 80% statistical power, an expected event rate(λ) of 1.24% derived from a previous study in the Arsi Zone [40], a critical tolerance limit(r) of one, a design effect of 1.5, and a 10% none-response rate. Statistical power refers to the probability of detecting a rare event if it truly exists, which is particularly relevant since esophageal cancer is a rare disease. The expected or mean event rate (λ) is calculated as the number of events (number of esophageal cancer patients) divided by the number of observations (number of kebele/woreda/zones). This represents the critical probability level above which the occurrence of cases is considered to indicate a cluster in the area. The Critical Event Limit (r) is the predetermined number of events that, if observed within the given sample size, leads to the conclusion that the expected event rate (λ) has been exceeded [45]. This yielded a required sample size of 216. However, since collecting information on all cases was essential for subsequent case-control and cohort studies, we included all 367 esophageal cancer patients who visited the four hospitals for diagnosis or treatment between April 3, 2019, and August 25, 2025 (Fig 2).

### Data collection

Socio-demographic and clinical data of esophageal cancer (EC) patients were collected from the cancer centers (clinics) of the selected hospitals through a review of patient charts and logbook. Patient files from the selected zones were identified using their unique medical record numbers. Data extraction was carried out by four trained nurses (one from each hospital) under the supervision of the principal investigator and two oncology residents working in the cancer treatment centers. The data extraction tool was developed based on a comprehensive literature review and information obtained from patient registration logbooks to capture relevant socio-demographic and clinical variables.

Following the chart review, the principal investigator contacted patients to verify their current health outcome for cases in which the outcome status was not recorded but tracing mechanisms were available. The kebele of each patient was then linked to Global Positioning System (GPS) coordinates using the Ethiopian shapefile obtained from the Ethiopian Space Science and Geospatial Institute (SSGI) to enable geographic mapping and identification of esophageal cancer clusters. Geocoding was performed by linking each patient’s recorded kebele to the corresponding kebele polygon in the Ethiopian shapefile. The geographic coordinates (latitude and longitude) for each patient were approximated as the centroid of their kebele of residence. Furthermore, the estimated population size of each kebele was obtained from the respective zonal health office.

### Operational definitions and measurements

**Clustering Variables** were Kebeles (level two), Districts (level three), and Zones (level four). **Spatial clustering** is a cluster in a geographical area if, within a certain period, that area has a higher percentage of its cases than the remaining geographical areas. The most likely cluster represents the geographic area where the observed number of esophageal cancer cases is significantly higher or lower than expected, showing the strongest statistical evidence of clustering**.** It corresponds to the zones, districts, or kebeles with the highest likelihood ratio (LLR), suggesting that the observed pattern is least likely to have occurred by chance under the null hypothesis of random distribution. Secondary clusters refer to other areas that also show statistically significant clustering but with lower likelihood ratios than the primary cluster. Likelihood Ratio (LLR) is calculated by Monte Carlo Simulation, defined by ${LLR=\left(\frac{c}{E\left[c\right]}\right)}^{c}{\left(\frac{C-c}{C-E\left[c\right]}\right)}^{C-c}I()$**,** where I() is an indicator function (inside =1, outside =0).

**Annual incidence rate of esophageal cancer** was calculated as the number of cases diagnosed per 100,000 populations per year using the formula:


$$\begin{array}{c} EC\text{ Annual incidence rate}=\frac{\text{Total cases}}{populationatrisk*Numberofyears}*\text{1}\text{0}\text{0},\text{0}\text{0}\text{0} \end{array}$$

**Risk (Observed/Expected)** is the observed number of cases within the cluster divided by the expected number of cases within the cluster when the null hypothesis is true, that is, when the risk is the same inside and outside the cluster. **Relative Risk** is the estimated risk within the cluster divided by the estimated risk outside the cluster. It is, calculated as $RelativeRisk(RR)=\frac{\text{Observed inside}/\text{Expected }inside}{\text{Observed outside}/\text{Expected outside }}$=$\frac{c/E(c)}{\left(C-c)/C-E(c\right)}$, and is used to measure how much higher (or lower) the risk of cases is inside a cluster compared to outside it. An RR = 1 indicates that the risk inside the cluster is equal to that outside (no elevated risk). An RR > 1 suggests a higher risk within the cluster, while a RR < 1 indicates a lower risk inside the cluster.

Where:


$$\begin{array}{c} =Observedcasesinsidethecluster, \end{array}$$

$$\begin{array}{c} E[c]=Expectedcasesinsidethecluster, \end{array}$$

$$\begin{array}{c} C=Totalobservedcases, \end{array}$$

$$\begin{array}{c} C-c=Observedcasesoutside, \end{array}$$

$$\begin{array}{c} C-E[c]=Expectedcasesoutside, \end{array}$$

**Gini index (coefficient),** calculated as $G=\frac{\sum_{i=\text{1}}^{n}(\text{2}i-n-\text{1})*x_{i}}{n*\sum_{i=\text{1}}^{n}x_{i}}$, was used to measure the inequality or concentration of esophageal cancer case across woredas or kebeles [46–48].

Where:


$$\begin{array}{c} \text{n}=\text{number of locations }(\text{or observations in the cluster}), \end{array}$$

$$\begin{array}{c} {\text{x}}_{\text{i}}=\text{number}\;\text{of}\;\text{cases}\;\text{in}\;\text{location}\;\text{i}\;(\text{sorted}\;\text{in}\;\text{ascending}\;\text{order}), \end{array}$$

$$\begin{array}{c} \text{i}=\text{The rank }(\text{position})\text{of location }{\text{x}}_{\text{i}}\text{ after sorting }(\text{smallest}=\text{i}=\text{1},\text{ largest}=\text{i}=\text{n}) \end{array}$$

The Gini coefficient provides a standardized, easily interpretable index that summarizes the degree of inequality in disease burden across geographic areas. The Gini coefficient is less sensitive to outliers and allows for comparison with other studies of spatial inequality in esophageal cancer outcomes.

**The Moran’s I,** calculated as $I=\frac{n}{W}*\frac{\sum_{i=\text{1}}^{n}\sum_{j=\text{1}}^{n}w_{ii}{(x}_{i}-\overset{―}{x})(x_{j}-\overset{―}{x})}{\sum_{i=\text{1}}^{n}{\left(x_{i}-\overset{―}{x}\right)}^{\text{2}}}$, was used to measure the spatial autocorrelation of esophageal cancer case across woredas or kebeles. It is employed to identify areas that exhibit potential hotspots, cold spots, or spatial outliers [49].

Where: $n=numberofspatialunits\left(\begin{array}{c} e.g.Districtor\\ Kebele \end{array}\right)$, $x_{i},x_{j}=Thevalueofthevariableatlocationiandj$, $\overset{―}{x}=Meanofthevariableoveralllocations$, $w_{ij}=Spatialweightbetweenunitsiandj$

(definesneighborsorproximity,usually 1 if areas are neighbors,0 otherwise), $W=sumofallspatialweights,W=\sum_{i}\sum_{j}w_{ij}$

A spatial weight matrix (W) was constructed to define neighborhood relationships using queen contiguity, meaning two kebeles were considered neighbors if they shared a common border or corner. Each kebele’s neighbors were identified using this method in GeoDa software [50].

Cases were all adult (≥ 18years) esophageal cancer patients residing in the five Zones that are endoscopically and/or histologically confirmed and attending the four hospital (i.e., Adama Hospital Medical College, Asela Teaching and Referral Hospital, Meda Welabu University General Hospital, and Tikur Anbessa Espetialized Hospital).

Latitude is Measured in degree, minute and second ((d^o^m’s” N/S), and entered as decimal number of degrees (DND) represents the north or south distance from the equator, and locations south of the equator was entered as negative numbers [51].

Longitude was measured in degree, minute and second(d^o^m’s” E/W), and entered as decimal number of degrees (DND) represents the east or west distance from the Prime Meridian (Greenwich, England), and locations west of the Prime Meridian was entered as negative numbers [51].

### Data processing and management

The data were collected using Kobo toolbox, and subsequently exported to SaTScan version 10.3.3 for cluster identification and estimation of magnitude of risks within the clusters [52]; GeoDa 1.22.01.21 assessing spatial autocorrelation and determining neighborhood relationship; and Quantum Geographic Information System (QGIS) desktop version 3.12 for spatial description and visualization. In addition, the data were imported in to R software version 4.3.3. for farther descriptive (Choropleth map), inferential and spatial inequality.

### Data analysis

Descriptive spatial analysis was employed to illustrate the spatial distribution of esophageal cancer cases using maps, plots, and summary statistics**.** This approach was used to visualize the distribution and rates of esophageal cancer across kebeles, districts, and zones**.**

**Fig 3:**
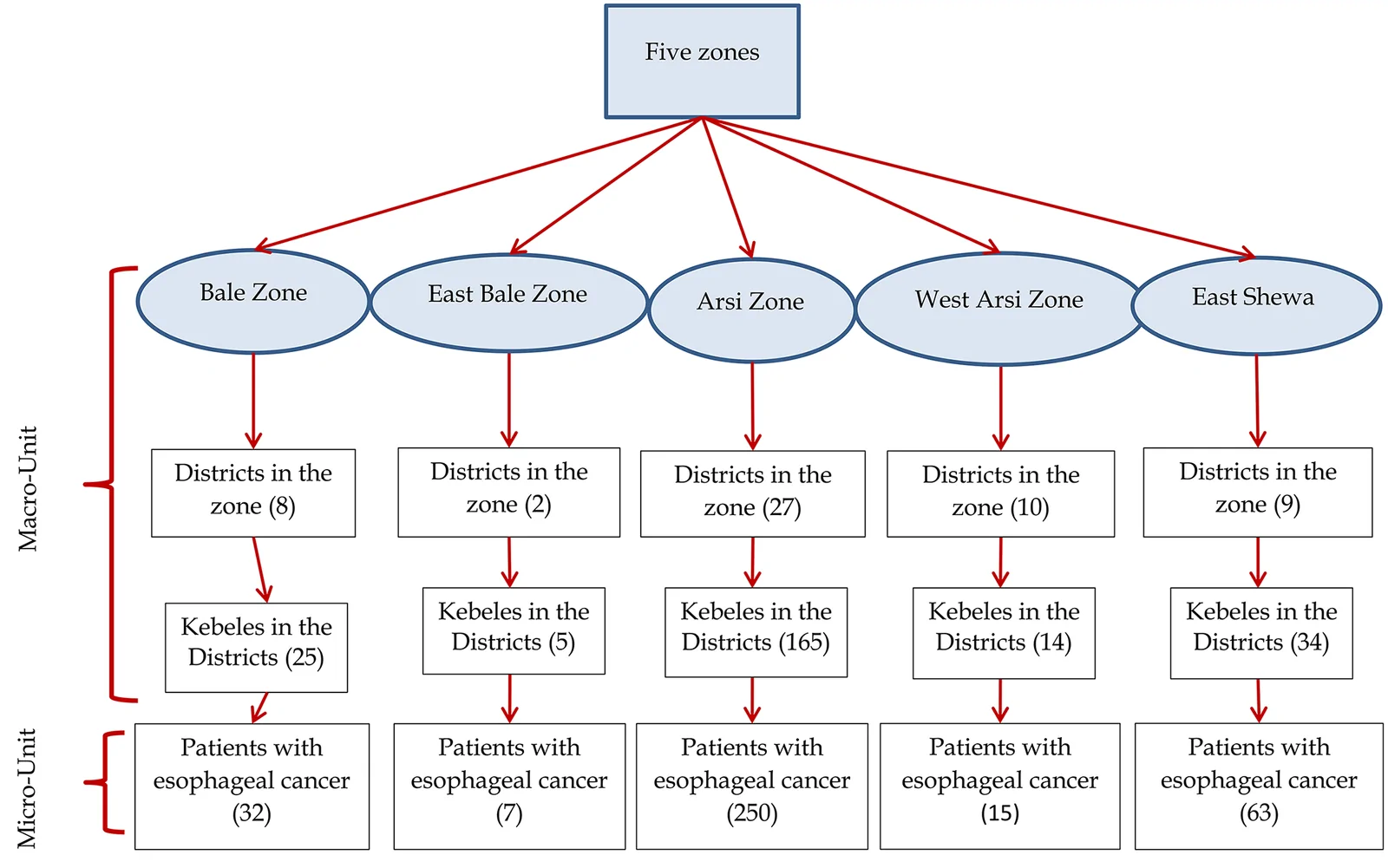
Depicting levels of spatial analysis of esophageal cancer in central Oromia, Ethiopia, 2025, n = 367.

The analysis was conducted across four hierarchical levels; patients with esophageal cancer (micro-unit), kebele, district, and zone (Fig 3). A circular scanning window of varying size was moved across the study area to identify clusters of esophageal cancer cases. The upper spatial cluster size was set to 50% of the total population at risk, as recommended by Kulldorff (2018), to enhance comparability across detected clusters. Statistical significance was assessed using 999 Monte Carlo replications [51,53]. Clusters exhibiting a high LLR and a p-value less than 0.05 were considered statistically significant high- or low-rate clusters. The discrete Poisson model was used to determine the magnitude of risk within the clusters and was interpreted using the risk ratio and its 95% confidence interval. Assessment of the Poisson distribution assumption at the Kebele level demonstrated a dispersion index of 1.06, indicating that the variance was approximately equal to the mean (mean = 1.51; variance = 1.61). Furthermore, the overdispersion test was not statistically significant (p = 0.441), providing no evidence of substantial overdispersion and supporting the suitability of the Poisson model for the spatial analysis.

Spatial inequality across the study area (woredas and kebeles) was assessed using the Gini index (coefficient)**.** A Gini value of zero indicates that esophageal cancer cases were evenly distributed, whereas a value approaching one signifies that most cases originated from a few locations within the cluster, reflecting inequality in case distribution**.** The Lorenz curve was employed to visualize the distributional inequality across districts and kebeles; curves lying below the line of equality denote greater disparities in case distribution. Additionally, bootstrapping was applied to estimate the average Gini coefficient and its 95% confidence interval**.**

Spatial autocorrelation of esophageal cancer cases was analyzed at both the kebele and woreda levels. Global Moran’s I was used to assess the overall spatial autocorrelation, while Local Moran’s I (Local Indicators of Spatial Association (LISA)) was applied to identify local clusters and outliers**.** LISA analysis was employed to detect hotspots, cold spots, and spatial outliers of esophageal cancer, specifically, whether woredas or kebeles with a high rate of esophageal cancer were surrounded by areas with similarly high rates (hotspots) or low rates (outliers), and conversely, whether those with low rates were surrounded by other low-rate areas (cold spots) or high-rate areas (outliers).

The magnitude of risk within the cluster was measured by

### Data quality assurance

The data extraction form was initially prepared in English, then translated into Amharic and Afaan Oromo, and subsequently back-translated into English by an independent language expert to ensure consistency. The principal investigators provided two days of training for data collectors and supervisors on data collection procedures using KoboToolbox**.**

Prior to the actual data collection, the tool was pretested on 10 (5%) esophageal cancer patient records from the selected hospitals to identify potential issues related to wording, interpretation, and compatibility with hospital record formats. Inter-rater reliability was assessed by having four independent data abstractors review the same 10 records. Fleiss’ Kappa was used to assess agreement for categorical variables, including sex, zone, residence, religion, type of diagnosis, diagnostic methods used, cancer stage, types of treatment provided, patients’ survival status, signs and symptoms, degree of tumor differentiation, tumor location, and histopathological subtype. The Intra-class Correlation Coefficient (ICC) was used to evaluate agreement for age of the patients.

Accordingly, the pretest demonstrated substantial to almost perfect agreement among the four raters. Fleiss’ Kappa values were 0.78 for sex (p < 0.001), 0.70 for zone (p < 0.001), 0.73 for residence (p < 0.001), 0.92 for religion (p < 0.001), 0.75 for type of patient diagnosed (p < 0.001), 0.89 for diagnostic methods used (p < 0.001), 0.91 for cancer stage (p < 0.001), 0.86 for type of treatment provided (p = 0.001), 0.77 for patients’ survival status (p < 0.001), 0.92 for signs and symptoms (p < 0.001), 0.87 for degree of tumor differentiation (p < 0.001), 0.91 for tumor location (p < 0.001), and 0.91 for histopathological subtype (p < 0.001).

The Intraclass Correlation Coefficient (ICC) for age was 0.99 (95% CI: 0.971–0.997; p < 0.001), indicating excellent consistency among the raters. The combined assessment of all categorical variables showed almost perfect agreement among the four abstractors, with a Fleiss’ Kappa value of 0.92 (p < 0.001). All discrepancies identified during the pretest were discussed and resolved, and the data abstraction tool was refined accordingly prior to the actual data collection.

Furthermore, the content and face validity of the data extraction form were evaluated prior to data collection. Content validity was assessed through an extensive review of relevant literature and expert evaluation by two specialists with experience in oncology and epidemiological research to ensure the relevance and comprehensiveness of the tool. Face validity was assessed by examining the feasibility, readability, consistency of formatting, and clarity of the language used in the data extraction form. Necessary revisions were made based on the feedback obtained. To maintain data quality, supervisors and investigators carried out daily cross-checking and provided regular supervision throughout the data collection process.

### Dissemination of findings

The final report will be presented at the annual conference of the universities, in addition to other local and worldwide conferences. The study’s findings will be presented to the Addis Abeba University Research and Community Service Office, the Arsi Zone Health Office, the West Arsi Zone Health Office, the East Shewa Zone Health Office, the Bale Zone Health Office, the East Bale Zone Health Office, and the Oromia Regional State Health Bureau. The findings will also be disseminated to the scientific community via publishing in national or international journals. Finally, the study’s findings will be presented to the school of public health, College of Health Science Addis Ababa University for defense in fulfillment of the requirements for the Ph.D. degree in Public Health.

### Ethics approval and consent to participate

Ethical approval was obtained from the Institutional Review Board (IRB) of the College of Health Sciences, Addis Ababa University (Protocol No. 088/24/SPH), as well as from the IRB of the Oromia Regional State Health Bureau (Protocol No. BFO/H2/032/17). In addition, administrative permission to access medical records was obtained from the respective authorities of the four participating hospitals prior to data collection. To ensure confidentiality and protect patient privacy, all personal identifiers, including names and phone numbers, were removed before data extraction, and only de-identified data were used for analysis. Access to the data was restricted to the research team, and all electronic data were stored on a password-protected computer belonging to the principal investigator. The study was conducted in accordance with relevant national guidelines and ethical principles governing research involving human participants. For patients whose survival status was not recorded and who were contacted through the tracing mechanism, verbal consent was obtained prior to data collection.

## Results

### Socio-demographic characteristics of esophageal cancer patients

The study included patients diagnosed between April 3, 2019, to August 25, 2025, across five zones, 56 districts and 243 Kebeles, with an average population of approximately 2,016,908. The study involved 367 esophageal cancer patients diagnosed within the aforementioned period.

The average age of the patients at diagnosis was 55.68 [95%CI: 54.45, 56.91] years. About sixty percent of the patients were females [60.22%; 95%CI: 54.99, 65.23%] while more than 80% of them were from rural [81.20%; 95%: 76.74, 84.99%].

The study showed that majority of the patients, 62.40% [95%CI: 57.20, 67.33%] were managed at Adama Hospital Medical College (AHMC). Geographically, most patients originated from the Arsi Zone, accounting for more than two-thirds of the total cases [68.12%; 95% CI: 63.05, 72.81] (Table 1).

**Table 1:** Sociodemographic characteristics of esophageal cancer patients, 2025 (n = 367).

Variables			Frequency (%)			95% CI of proportion (%)
**Hospitals**						
Adama (AHMC)			229 (62.40)			57.20, 67.33
Asella (ATRH)			82 (22.34)			18.26, 27.02
Goba (GRH)			36 (9.81)			7.07, 13.43
Tikur Anbesa (TASH)			20 (5.45)			3.45, 8.43
**Gender**						
Male			146 (39.78)			34.77, 45.00
Female			221 (60.22)			54.99, 65.23
**Residence**						
Rural			298 (81.20)			76.74, 84.99
Urban			69 (18.80)			15.01, 23.26
**Religion**						
Muslim			250 (68.12)			63.05, 72.81
Orthodox			89 (24.25)			20.02, 29.03
Protestant			19 (5.18)			3.23, 8.11
Wakefata			9 (2.45)			1.20, 4.77
**Patient’s Zone**						
Arsi			250 (68.12)			63.05, 72.81
Bale			32 (8.72)			6.13, 12.21
East Bale			7 (1.91)			0.84, 4.06
East Shewa			63 (17.17)			13.53, 21.51
West Arsi			15 (4.08)			2.39, 6.80
**Age at diagnosis**						
**Range**	**1**^**st**^ **quart**	**Mean**	**Median**	**SD**	**3**^**rd**^ **quart**	95%CI of mean
18-95	50.0	55.68	56.0	11.95	65.0	54.45, 56.91

### Clinical characteristics of esophageal cancer patients

Most patients were newly diagnosed [94%; 95%CI: 90.93, 96.12%], with only a small proportion being relapse, recurrent, or transferred cases. Case confirmation diagnosis was predominantly made through endoscopy [93%; 95%CI: 89.66, 95.23%] and CT scans [92%; 95%CI: 88.73, 94.56%], while clinical diagnosis was less common [13%; 95%CI: 9.87, 17.06%]. About one-third had histological confirmation [29.43%; 95%CI: 24.87, 34.43].

More than two third of the patients [69.76%; 95%CI: 64.73, 74.36%] presented with late stages (III-IV) cancer, and only few patients were diagnosed in early stages (I–II) [5.72%; 95%CI: 3.66, 8.75%]. For one forth of the patients the cancer stages were not recorded [24.92%; 95%CI: 20.27; 29.32%]. Chemotherapy was the most frequently provided treatment [93.19%; 95%CI: 89.98, 95.46%], whereas surgery [10.35%; 95%CI: 7.07, 13.43%] and palliative care [3%; 95%CI: 1.58, 5.46] were less commonly administered.

Regarding survival status, 69.48% [95%CI: 64.45, 74.10] of patients were alive at the time of record review, while nearly one third of the patients [29.16%; 95%CI: 24.61, 34.14%] had unknown survival status.

Dysphagia was the most commonly reported symptom [87.47%; 95%CI: 83.54, 90.59%], followed by weight loss [25.61%; 95%CI: 21.29, 30.46]. Tumor differentiation status was largely unrecorded [74.11%; 95%CI: 69.26, 78.46], but among documented cases, well-differentiated tumors were most frequent [15.80%; 95%CI: 12.31, 30.04].

The lower thoracic esophagus was the most frequent tumor location [51.50%; 95%CI: 46.26, 56.71%], followed by the middle thoracic tumor [18.80%; 95%CI: 15.01, 23.26%] and the gastroesophageal junction [19.07%; 95%CI: 15.26, 23.55]. Histopathologically, squamous cell carcinoma was the predominant subtype [72.48%; 95%CI: 67.55, 76.93%], with adenocarcinoma accounting for 19.07% [95%CI: 15.24, 23.55] (Table 2).

**Table 2:** Clinical characteristics of esophageal cancer patients in Central Oromia, Ethiopia, 2025 (n = 367).

Variables	Frequency (%)	95% CI of %
**Types of patients diagnosed**		
New	345 (94.0)	90.93, 96.12
Relapse on follow up	6 (1.64)	0.67, 3.70
Relapse/recurrence (RR)	3 (0.82)	0.21, 2.57
Transferred in	13 (3.54)	1.98, 6.13
**Diagnosis performed**		
Clinical	48 (13.08)	9.87, 17.06
Endoscopy	341 (92.92)	89.66, 95.23
Histology	108 (29.43)	24.87, 34.43
X-ray (barium swallow)	65 (17.71)	14.02, 22.09
CT-scan	338 (92.10)	88.73, 94.56
Ultrasound	193 (52.59)	47.34, 57.78
**Cancer stages**		
Stage I	6 (1.65)	0.67, 3.70
Stage II	15 (4.09)	2.39, 6.80
Stage III	58 (15.80)	12.37, 20.04
Stage IV	198 (53.95)	48.70, 59.12
Not recorded	90 (24.92)	20.27, 29.32
**Type of treatment given**		
Surgery	38 (10.35)	7.52, 14.05
Palliative care	11 (3.0)	1.58, 5.46
Chemotherapy	342 (93.19)	89.98, 95.46
Not recorded	9 (2.45)	1.20, 4.77
**Patients survival status**		
Alive	255 (69.48)	64.45, 74.10
Death	5 (1.36)	0.50, 3.34
Survival status unknown	107 (29.16)	24.61, 34.14
**Sign and symptom**		
Dysphagia	321 (87.47)	83.54, 90.59
Weight loss	94 (25.61)	21.29, 30.46
Vomiting	62 (16.90)	13.29, 21.21
Not recorded	27 (7.36)	4.99, 10.65
**Degree of tumor differentiation**		
Well differentiated	58 (15.80)	12.31, 20.04
Moderately differentiated	7 (1.91)	0.84, 4.06
Poorly differentiated	17 (4.63)	2.81, 7.46
Not conclusive	13 (3.54)	1.98, 6.13
Not recorded	272 (74.11)	69.26, 78.46
**Tumor location**		
Cervical	7 (1.91)	0.84, 4.06
Upper thoracic	31 (8.45)	5.90, 11.90
Middle thoracic	69 (18.80)	15.01, 23.26
Lower thoracic	189 (51.50)	46.26, 56.71
Gastroesophageal junction	70 (19.07)	15.26, 23.55
Not recorded	10 (2.73)	1.39, 5.12
**Histopathological sub-type**		
Squamous cell carcinoma	266 (72.48)	67.55, 76.93
Adenocarcinoma	70 (19.07)	15.24, 23.55
Adeno-squamous cell carcinoma	3 (0.82)	0.21, 2.57
Not specified	28 (7.63)	5.22, 10.96

### District- and kebele-level esophageal cancer case rates and counts

At the district level, the mean case count was 6.55 (±7.30), with Robe District recording the maximum (42 cases). Siraro District had the highest district-level case rate (104.66 per 100,000), compared to the average of 19.39 (±21.20) per 100,000.

At the kebele level, the average case count was 1.51 (±1.27), with Robe 01 reporting the highest number of cases (14). Kulla 01 had the highest case rate at 179 per 100,000, far above the kebele average of 22.67 (±15) per 100,000.

**Fig 4:**
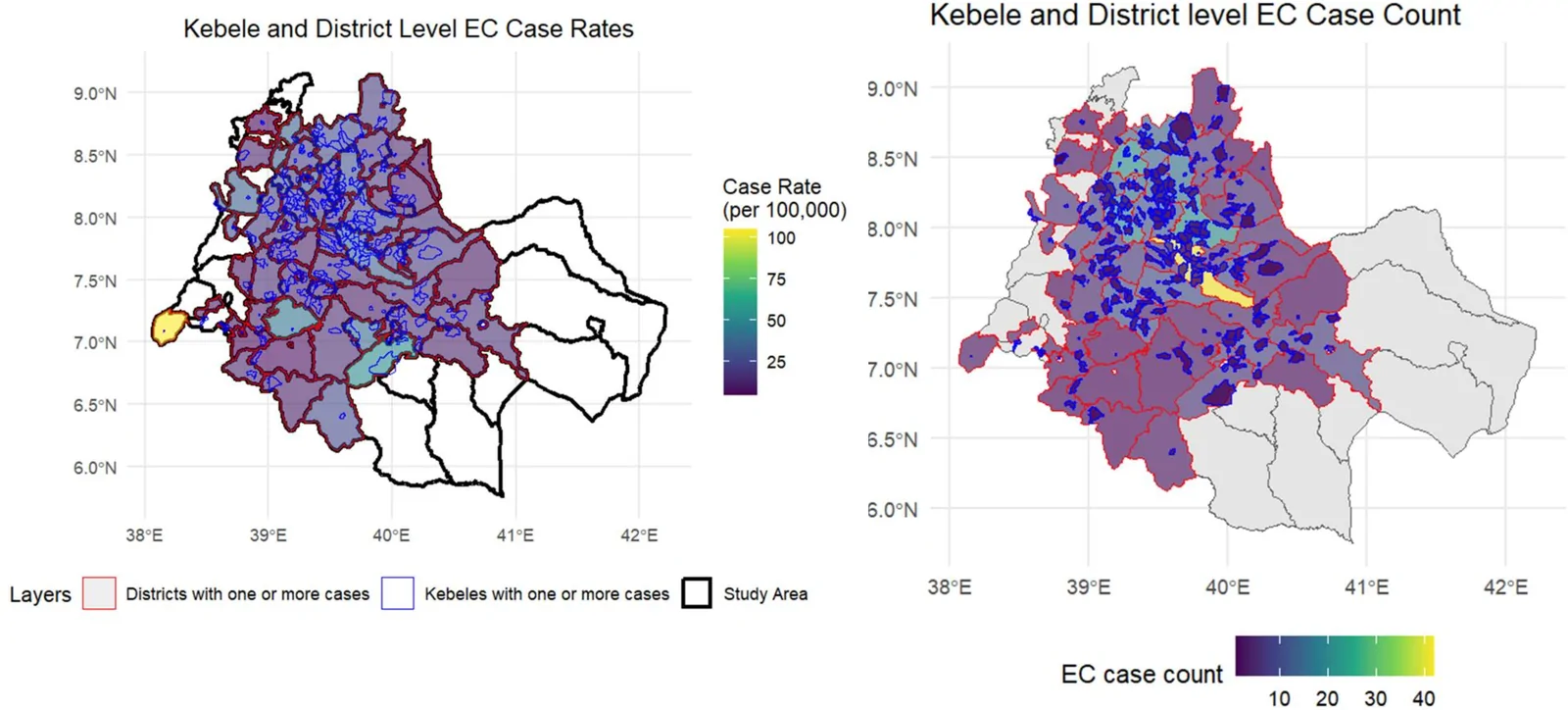
A choropleth map showing the distribution of esophageal cancer cases in the study area based on case rates and counts (n = 367).

Most of the districts and kebeles included in the study were located in Arsi Zone, which accounted for 48.21% [95%CI: 34.84, 61.83] of districts and 67.90% [95%CI: 61.58, 73.65] of kebeles. Other zones such as Bale, East Bale, East Shewa, and West Arsi contributed smaller proportions (Table 3 and Fig 4).

**Table 3:** Esophageal cancer case count and rate at Kebele and Wored level, 2025 (n = 367).

District Level (n=56distiricts)			
**Characteristics**	**Average/sd**	**Max**	**Name of District with max. value**
Case count	6.55 (7.30)	42	Robe district
Case rate per 100, 000	2.77 (2.14)	14.95	Siraro district
**Kebele level (n= 243 kebeles)**			
**Characteristics**	**Average/sd**	**Max**	**Number of kebele with Max. value**
Case count	1.51 (1.27)	14	Robe 01
Case rate per 100, 000	3.25 (3.05)	25.65	Kulla 01
**Number of district in the zones (56)**			
Zones	Frequency (%)		**95% CI of %**
Arsi	27 (14.21)		34.84, 61.83
Bale	8 (14.29)		6.80, 26.78
East Bale	2 (3.57)		0.62, 13.38
East Shewa	9 (16.07)		8.05, 28.83
West Arsi	10 (17.86)		9.34, 30.85
**Number of kebeles in the zones (243)**			
Zones	Frequency (%)		**95% CI of %**
Arsi	165 (67.90)		61.58, 73.65
Bale	25 (10.29)		6.90, 14.98
East Bale	5 (2.06)		0.76, 5.01
East Shewa	34 (13.99)		10.01, 19.14
West Arsi	14 (5.76)		3.31, 9.69

### Spatial inequality of esophageal cancer

**Fig 5:**
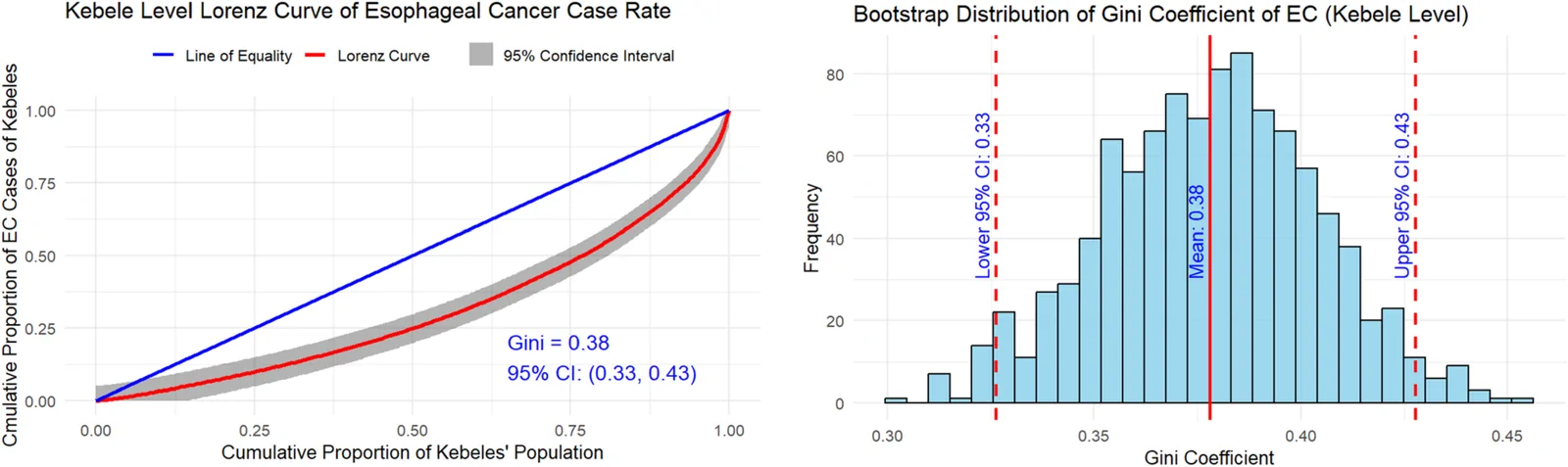
Bootstrap and Lerenz curve depicting the spatial inequality of esophageal cancer at kebele level, n = 367.

**Fig 6:**
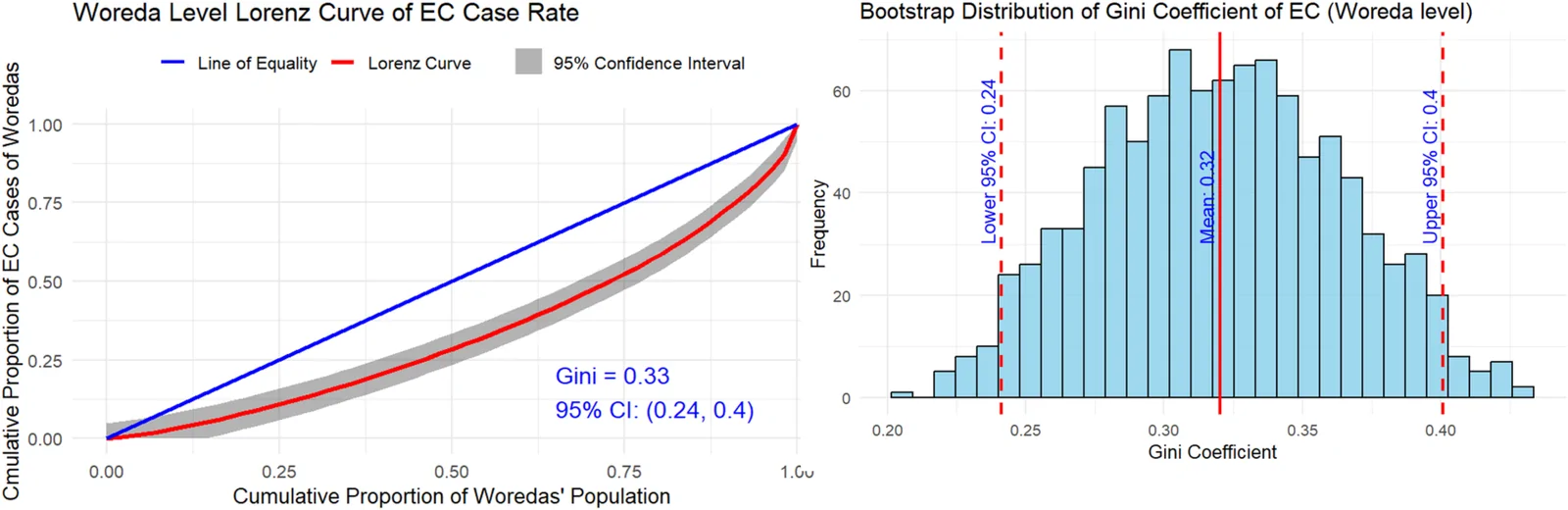
Bootstrap and Lerenz curve depicting the spatial inequality of esophageal cancer at district level, n = 367.

The calculated Gini coefficients indicated a statistically significant spatial inequality in the distribution of esophageal cancer (EC) cases within the study area. Specifically, the Gini coefficient at the Kebele level was 0.38 [95% CI: 0.33, 0.43] (Fig 5)**,** while at the Woreda level it was 0.33 [95% CI: 0.24, 0.40] (Fig 6)**.** These values demonstrate that EC cases were not evenly distributed across administrative units. Instead, a relatively small number of Kebeles and Woredas accounted for a disproportionately large share of the total EC cases.

### Spatial Autocorrelation of esophageal cancer

At the woreda and kebele levels, the spatial weights matrices showed that the number of neighboring woredas ranged from 0 to 9, and the number of neighboring kebeles ranged from 0 to 6, with means of 4.46 (±0.10) and 1.14 (±0.09), respectively. Notably, at least one woreda and one kebele had no adjacent neighbors, indicating spatial isolation at both levels. The proportion of non-zero connections was 7.97% for woredas and 0.47% for kebeles, reflecting a relatively sparse spatial structure.

At woreda level the global Moran’s I showed there was no spatial autocorrelation (I = 0.13; p-value = 0.075) in the distribution of esophageal cancer cases across the study area at the 5% level. However, at the kebele level, the analysis revealed a statistically significant positive spatial autocorrelation (I = 0.17; p-value = 0.037). However, at the district (woreda) and kebele level, the Local Indicators of Spatial Association (LISA) analysis demonstrated a significant spatial autocorrelation of esophageal cancer cases across the study area. In the East Shewa Zone, Adama Town (Local Moran’s I = 0.32; p-value = 0.045) and Adama District (Local Moran’s I = 0.08; p-value = 0.045) formed high–high clusters, indicating hotspots where districts with high case rates were surrounded by others with similarly high rates. Conversely, in the West Arsi Zone, Shashemene (Local Moran’s I = 1.34; p-value = 0.038) and Kokosa District (Local Moran’s I = 0.60; p-value = 0.002) exhibited low–low clustering, representing cold spots with low case rates neighboring other low-rate areas. Meanwhile, Liben Chukala (Low–High; Local Moran’s I = −1.06; p-value = 0.041) and Gedeb Asesa (High–Low; Local Moran’s I = −2.16; p-value = 0.007) were identified as spatial outliers, indicating that their case rates contrast with those of adjacent districts.

**Fig 7:**
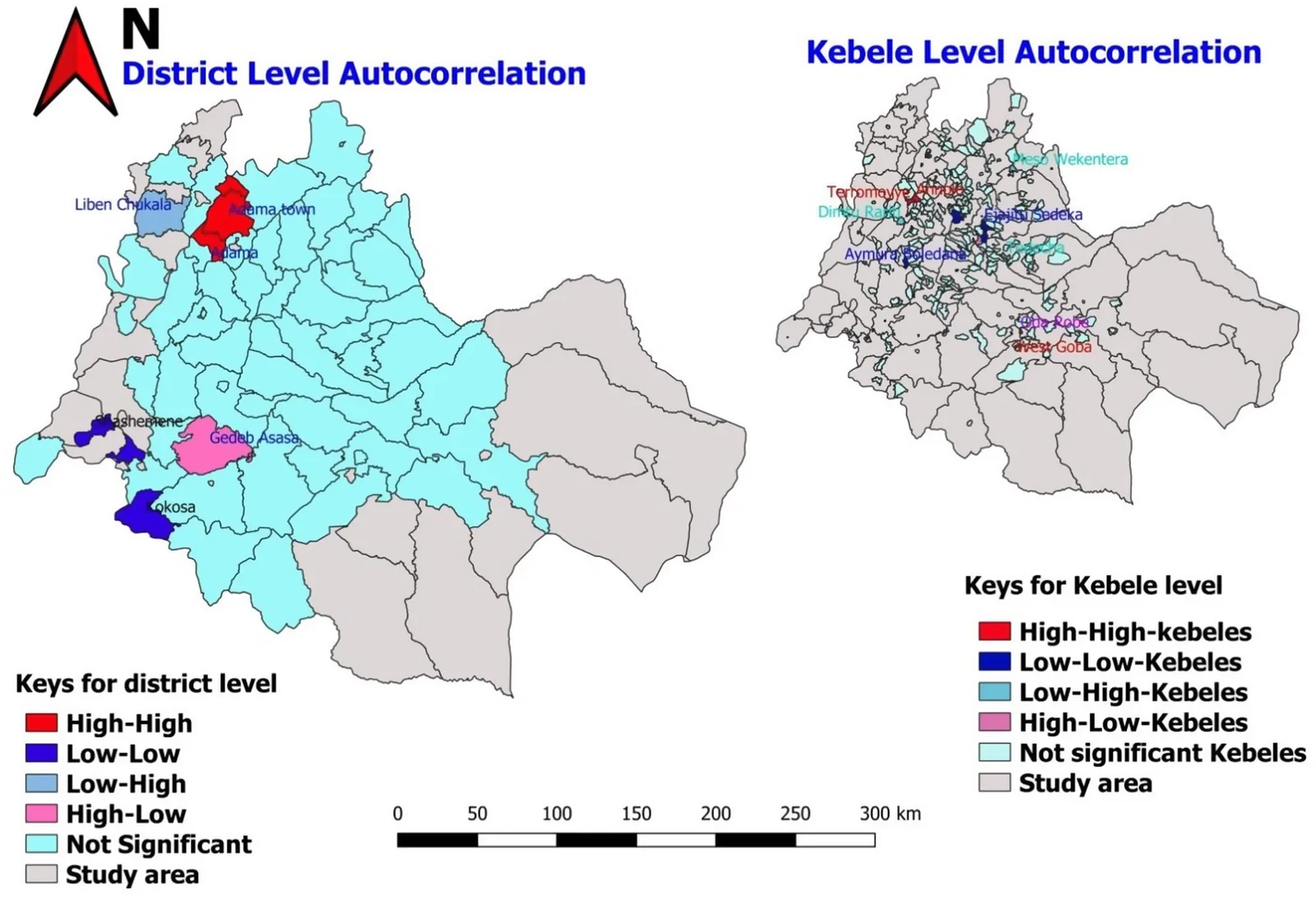
District and Kebele-Level Spatial Autocorrelation of Esophageal Cancer (n = 367).

At kebele level, West Goba (Bale Zone**,** Goba town) was identified as a strong hotspot (high–high cluster) (Local Moran’s = 7.78; p-value = 0.011)**,** while Oda Robe (Robe Town) appeared as a spatial outlier (high–low) (Local Moran’s I = −0.003; p-value = 0.023). In the Arsi Zone**,** Annole and Terromoyye showed high–high clusters**,** indicating local hotspots, whereas several kebeles such as Aymura Boledana**,** Asandabo**,** Tulu Bego**,** Marena Gerjela**,** Ejajitu Sedeka**,** and Kara Lencha formed low–low clusters, representing cold spots. Other kebeles including Sude Walte**,** Dimtu Rariti**,** Meso Wekentera**,** and Tedecha showed high–low or low–high patterns, indicating spatial outliers (Table 4 and Fig 7).

**Table 4:** Depiction of Global and Local Spatial Autocorrelation of Esophageal Cancer Cases in the Study Area (n = 367).

District level global test (n=56)					
Ob. Moran’s I	Exp. Moran’s I	Mean	Var. Moran’s I	Z-score	P-value
0.13	-0.02	-0.02	0.10	1.55	0.0750
Neighborhood					
Min. neighbors	Max. neighbors	Mean neighbors	Median neighbors		% non-zero
0.0	9.0	4.46	5.0		7.97
Kebele level global test					
Ob. Moran’s I	Exp. Moran’s I	Mean	Var. Moran’s I	Z-score	P-value
0.17	-0.01	-0.01	0.09	2.08	0.037
Neighborhood					
Min. neighbors	Max. neighbors	Mean neighbors	Median neighbors		% non-zero
0	6	1.14	1.0		0.47
**District level Local Indicators of Spatial Association (LISA)**					
**Zones**	**District**		**LISA Cluster**	**Local Moran’s I**	**P-value**
East Shewa	Adama District		High-High	0.08	0.045
	Adama Town		High-High	0.32	0.045
	Liben Chukala District		Low-High	-1.06	0.041
West Arsi	Shashemene District		Low-Low	1.34	0.038
	Kokosa District		Low-Low	0.60	0.002
	Gedeb Asesa		High-Low	-2.16	0.007
**Kebele level Local Indicators of Spatial Association (LISA)**					
**Zones**	**District**	**Kebeles**	**LISA Cluster**	**Local Moran’s I**	**P-value**
Bale	Goba	West Goba	High-High	7.78	0.011
	Robe Town	Oda Robe	High-Low	-0.003	0.023
Arsi	Hitosa	Annole	High-High	0.50	0.031
		Terromoyye	High-High	1.26	0.047
	Digaluna Tijo	Aymura Boledana	Low-Low	0.46	0.023
	Robe	Asandabo	Low-Low	0.57	0.026
		Sude Walte	High-Low	-0.16	0.001
	Lodehitosa	Tulu Bego	Low-Low	0.68	0.038
	Sude	Marena Gerjela	Low-Low	0.52	0.024
		Ejajitu Sedeka	Low-Low	0.41	0.015
	Diksis	Kara Lencha	Low-Low	0.81	0.05
	Zewaydugda	Dimtu Rariti	Low-High	-0.34	0.042
	Guna	Meso Wekentera	Low-High	-1.48	0.039
	Amigna	Tedecha	Low-High	-0.19	0.014

### Risk of esophageal cancer within the cluster windows

**Fig 8:**
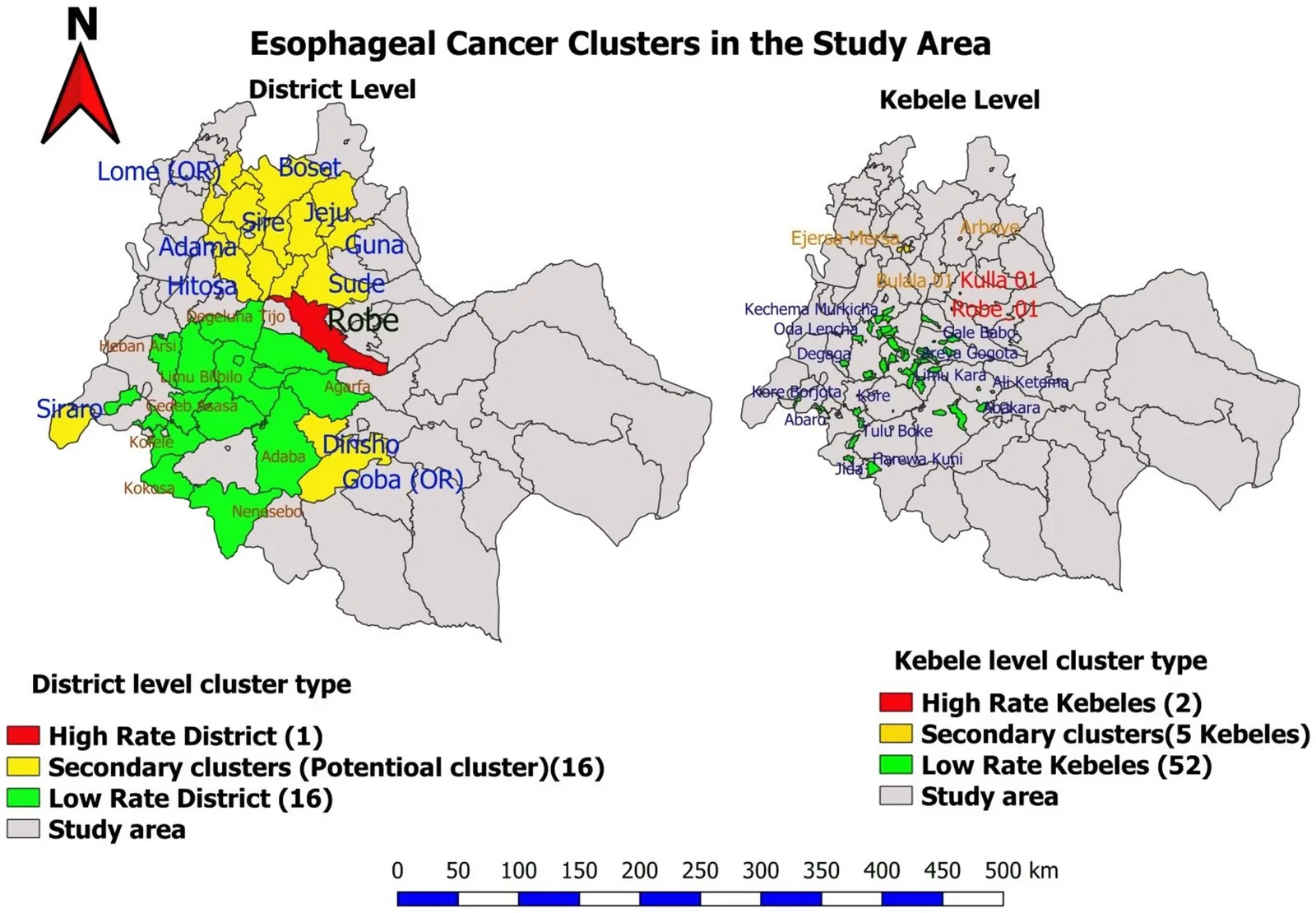
Spatial Distribution of High/Low Rates and Potential Clusters of Esophageal Cancer across Districts and Kebeles (n = 367).

A total of 367 esophageal cancer cases were reported in the study area, corresponding to an annual incidence rate of 2.6 [95%CI: 2.34, 2.88] cases per 100,000 populations. At district level Robe district was identified as a high-risk area for esophageal cancer. The population in Robe district had nearly twice the risk of developing esophageal cancer compared to populations outside this district [RR = 1.93; 95%CI: 1.43, 261; LLR = 6.82; p-value = 0.022; cluster center/ radius: (7.691000^o^ N, 39.801000^o^ E)/ 0 km]. A statistically significant low-risk cluster of esophageal cancer was identified across 17 districts. The annual incidence in this cluster was 1.6 cases per 100,000 populations. The population in these districts had about half the risk of developing esophageal cancer compared to populations outside the districts [RR = 0.55; 95 CI: 1.81, 2.10; LLR = 9.31; p-value = 0.0051; cluster center/ radius: (7.183000^o^ N, 39.173000^o^ E)/ 69.03 km] (Table 5 and Fig 8).

**Table 5:** SaTScan output depicting the risk of esophageal cancer within the identified cluster window of districts (n = 367).

Location/ cluster number	Gini cluster/ type	Observed	Expected	Annual case per 100,000	O/E	RR (95%CI)	LLR	P-value
Robe district/ 1	Yes/ primary	42	23.01	4.7 (3.42, 6.42)	1.83	1.93 (1.43, 2.61)	6.82	0.022
Cluster two (11 districts)*	No/ Secondary	170	146.30	3.0 (2.58, 3.51)	1.16	1.30 (0.12, 1.51)	3.15	0.630
Goba and Dinsho/ 3	No/ Secondary	8	3.09	6.7 (2.90, 13.25)	2.59	2.62 (0.30, 5.28)	2.73	0.763
Dodota district/4	No/ Secondary	16	9.52	4.4 (2.50, 7.09)	1.68	1.71 (0.04, 2.82)	1.88	0.950
Siraro/ 5	No/ Secondary	2	0.35	14.9 (1.80, 54.01)	5.75	5.78 (0.40, 6.46)	1.85	0.954
Jeju/ 6	No/ Secondary	16	10.11	4.1 (2.35, 6.68)	1.58	1.61 (0.98, 2.67)	1.50	0.994
Low rate (17 districts)**	Yes/ primary	54	87.38	1.60 (1.81, 2.10)	0.62	0.55 (0.41, 0.73)	9.31	0.0051

* Sire, Lude Hitosa, Diksis, Adama town, Adama, Hitosa, Boset, Merti, Guna, Sude, Lome (OR)

** Gedeb Asasa, Kore, Limu Bilbilo, Dodola, Bekoji town, Inkolo Wabe, Kofele, Munessa, Adaba, Heban Arsi, Kokosa, Shirka, Shashemene, Degeluna Tijo, Nenesebo, Dinsho, Agarfa.

At Kebele-level a strong spatial cluster of esophageal cancer was identified. Robe 01 Kebele showed an almost eight-fold elevated risk of esophageal cancer relative to neighboring kebeles [RR = 7.8; 95% CI: 4.57, 13.31; LLR = 16.35; p-value = 0.000024, cluster center/ radius: (7.862324^o^ N, 39.625791^o^ E)/ 0 km] Similarly, Kula 01 kebele showed approximately a ten-fold increased risk compared to surrounding kebeles [RR = 9.96; 95% CI: 4.45, 22.32; LLR = 8.35; p-value = 0.034; cluster center/ radius: (7.976705^o^ N, 39.688567^o^ E)/ 0 km]. In contrast, a large low-rate cluster composed of 52 kebeles demonstrated a significantly reduced risk of esophageal cancer. The population in these kebeles had about half the risk of developing esophageal cancer compared to populations outside the kebeles [RR = 0.55; 95% CI: 0.41, 0.73; LLR = .87; p-value = 0.014; cluster center/ radius: (7.103042^o^ N, 39.190783^o^ E)/ 83.03 km] (Table 6 and Fig 8).

**Table 6:** SaTScan output depicting the risk of esophageal cancer within the identified cluster window of Kebeles (n = 367).

Location/ cluster number	Gini cluster/ type/	Observed	Expected	Annual case per 100,000	O/E	RR (95%CI)	LLR	P-value
Robe 01/ 1	Yes/Primary	14	1.86	19.7 (10.78, 33.09)	7.54	7.8 (4.57, 13.31)	16.35	0.000024
Kula 01/ 2	Yes/ Secondary	6	0.61	25.6 (9.41, 55.82)	9.81	9.96 (4.45, 22.32)	8.35	0.034
Bulala 01/3	No/ Secondary	3	0.35	22.4 (4.62, 65.50)	8.57	8.64 (0.77, 26.91)	3.81	0.865
Arboye/ 4	No/ Secondary	6	1.56	10.1(3.90, 21.92)	3.85	3.90 (0.74, 8.74)	3.67	0.889
Ejersa Mersa, Tedechaguracha, Dera 01/ 6	No/ Secondary	9	3.58	6.6 (3.01, 12.48)	2.51	2.55 (0.32, 4.94)	2.92	0.987
Low rate (52 kebeles)*	Yes/ primary	56	90.86	1.6 (1.23, 2.09)	0.62	0.55 (0.41, 0.73)	9.87	0.014

*01 Kebele (Gedeb Asesa), Tulu Boke, Inkolo_gerjeda, Tulu Kore, Gadisa_derara, Kore, Waji Asharge, Farichu, Meraro, Sharo Kerensa, Abamo, Koji_kaka, Limu Kara, Gafarisa Kara, Koma_walkite, Kofele 01, Ciba_mikael, Limu Lema, Bekoji 01, Mecitu Lema, Harewa Kuni, Bekoji_nnegoso, Areya Gogota, Jida, Gerenba Dima, Lemu Guna, Mitana Gado, Lemu_mikael, Degaga, Limu Ena Tijo, Abaro, Ego 01, Ebicha, Aymura Boledana, Gusha Timela, Gobess_02, Oda Lencha, Birbo And Cole, Gobessa_01, Ashebeka Welkite, Gale Babo, Lole Abosera, Agerfa Ketema, Sagure 01, Abakara, Burkitu Alkesa, Hela Galabe Amaja, Kechema Murkicha, Kore Borjota, Sole Oda, Shala_cabiti, Ali Ketema.

## Discussion

This identified noticeable geographic heterogeneity of esophageal cancer at both the woreda and kebele levels in central Oromia. The Gini coefficients indicated statistically significant inequality, demonstrating that EC cases were unevenly distributed across administrative units. A relatively small number of kebeles and woredas contributed a disproportionately large proportion of the total cases. The Local Moran’s I revealed significant spatial clustering at both the woreda and kebele levels. Furthermore, spatial scan statistics confirmed the presence of both high-risk and low-risk clusters within the study area.

Local spatial autocorrelation (LISA) provided further evidence of small-area spatial autocorrelation. Several woredas, including Adama Town and Adama District, exhibited significant high–high clusters, indicating consistent hotspots of elevated EC burden. Conversely, Shashemene and Kokosa formed significant low–low clusters, reflecting areas with low case rates. This implies that clustering becomes more detectable at finer geographical scales, consistent with the notion that localized determinants, such as environmental exposures, dietary practices, or access to diagnostic services may operate at the community level [27,29,42,54]. The presence of spatial outliers, such as Liben Chukala (low–high) and Gedeb Asasa (high–low), highlights districts whose EC patterns differ markedly from their neighbors. These findings emphasize that EC distribution is not random but influenced by localized factors that may vary sharply between adjacent areas [27,28]. Previous study from China demonstrated substantial geographic variation in esophageal cancer rates over relatively short geographic distances [29].

At the kebele level, spatial heterogeneity was even more pronounced. Robe 01 and Kula 01 emerged as strong cluster with markedly elevated relative risks, nearly eight-fold and ten-fold higher than their surrounding kebeles, respectively. Additional high–high clusters were identified in West Goba, Annole, and Terromoyye. In contrast, 52 kebeles formed a large low-rate cluster with significantly reduced risk, indicating considerable geographic variability within short distances. The identification of hot and cold spots at this fine spatial resolution highlights the importance of kebele-level analysis for uncovering micro-area variations that may be obscured at broader administrative scales [42]. This approach is also crucial for accurately identifying local-level risk. These findings can guide future studies to explore localized risk factors, thereby enhancing efforts to reduce the incidence of esophageal cancer [49].

The spatial scan statistics further demonstrated that both high-risk and low-risk clusters exist in the study area. Robe district was identified as a high-risk woreda, with nearly double the risk of EC compared to areas outside the cluster. Meanwhile, a large low-risk cluster covering 17 districts had significantly reduced incidence. At the kebele level, the magnitude of risk was even more extreme, with risk estimates reaching nearly ten-fold in hotspot areas. These findings collectively suggest that EC in Central Oromia is characterized by strong geographic disparities, with specific communities facing substantially higher risk than others. This finding is consistent with other studies showing that esophageal cancer varies across geographic areas [40,49,54].

The observed clustering is in agreement with findings from the East African esophageal cancer corridor, where localized high-incidence areas of esophageal cancer have been reported in Ethiopia, especially in the Bale and Arsi Zones, as well as in other countries, including Kenya, Tanzania and Malawi [55,56]. Several contextual factors may help explain these patterns. Previous studies in Ethiopia and East Africa have linked EC risk to consumption of very hot beverages [41,57], lack of fruits and vegetables [28,57,58], behavioral risk factors [58], poor oral health [57], and environmental carcinogens such as polycyclic aromatic hydrocarbons (PAHs) [41,57]. The strong clustering observed in particular kebeles suggests that local environmental or behavioral factors may be disproportionately concentrated in certain communities. In addition, limited access to early diagnosis may contribute to spatial inequality, as remote areas with fewer health facilities may experience delayed detection and reporting.

The study was limited to the variables available in the logbooks and patients’ charts, which excluded important clinical or socio-demographic factors that were not routinely recorded, as well as individuals with incomplete information. Referral bias may have influenced our results, as patients referred to the four study hospitals may differ systematically from those preferred elsewhere. Geocoding was based on kebele centroids rather than exact GPS coordinates which may introduce positional inaccuracies and affect cluster boundary precision. Furthermore, the spatial analysis limits causal inference, as individual-level risk factors could not be assessed. Environmental exposures that could explain the observed clustering were also not measured. Despite these limitations, the study offers robust evidence of spatial inequality and localized clustering.

In conclusion, this study identified marked spatial heterogeneity of esophageal cancer across Arsi, West Arsi, Bale, East Bale, and East Shewa zones in central Oromia. Significant clustering was observed at both woreda and kebele levels. Robe district had nearly twice the risk, Robe 01 kebele had more than seven times the risk, and Kula 01 kebele had nearly ten times the risk compared to other areas. Based on these findings, we recommend prioritizing Robe district and Robe 01 and Kula 01 kebeles for targeted esophageal cancer screening, particularly for age group 30 years and older; allocating diagnostic resources to health facilities serving these high-risk districts and kebeles; and implementing community health education on modifiable risk factors in the study area. Future research should investigate individual-level risk factors to explain the cause of observed clustering patterns.

## Abbreviations

AC: Adenocarcinoma
ESCC: Esophageal squamous cell carcinoma
GIS: Geographic information system
GPS: Global Positioning System
SCC: Squamous cell carcinoma
LISA: Local indicators of Spatial Association
