# Supplementary material for: Spatial clustering of esophageal cancer in central Oromia, Ethiopia: A spatial autocorrelation and scan statistics analysis
Source: PLoS One. 2026 Sep 15;21(9):e0357042. doi: 10.1371/journal.pone.0357042 (PMC13577588; doi:10.1371/journal.pone.0357042)
Supplement: Supplementary file 1 [file pone.0357042.s001.docx]

**1. Title**

**Line 1**

- The title is clear but could be improved by **indicating the analytical methods** (e.g., spatial scan statistics, Moran’s I).
- Consider specifying **study period** if applicable.
- Suggested revision example:
  *“Spatial Clustering of Esophageal Cancer in Central Oromia, Ethiopia: A Spatial Autocorrelation and Scan Statistics Analysis.”*

**2. Abstract**

**Background**

**Line 6–9**

- The sentence structure is unclear and grammatically inconsistent.
- “GLOBOCAN report showed” should be written as **“According to the GLOBOCAN 2022 report”**.
- The statement **“with the two zones having the highest prevalence (68.3%)”** needs a **clear citation or explanation of the calculation method**.
- “Undertaking thorough study” should be **“a thorough study”**.

Reviewer concern:

- The background mixes **global statistics and regional statistics without clear transition**.

**Objective**

**Line 12**

- The objective is clear but **too broad**.
- Consider specifying **methodological approach**.

Example improvement:

“To assess spatial clustering and identify geographic hotspots of esophageal cancer in Central Oromia using spatial autocorrelation and spatial scan statistics.”

**Methods**

**Line 15–21**

Issues:

1. **Study design**
   - “The cross-sectional study design was employed” should specify **retrospective record review**.
2. **Sample size**
   - “367 patients collected from records” should be clarified:
     - What **years** were included?
     - Were cases **confirmed histologically**?
3. **Hospital names**
   - Ensure correct spelling:
   - “Tikur Anmbassa” → **Tikur Anbessa**
4. **Geographic variables**
   - You mention **kebele, woreda, and zone**, but it is unclear:
     - Which level was used in the analysis?
5. **Spatial data source**
   - The Ethiopian Space Science and Geospatial Institute should be cited properly:
   - Ethiopian Space Science and Geospatial Institute

**Statistical methods**

The abstract mentions:

- Gini coefficient
- Moran’s I
- Relative risk
- Log likelihood ratio

Reviewer concern:

- The **software used (SaTScan, ArcGIS, R)** should be mentioned.

**Results**

Common issues observed in the manuscript style:

- Results must include **numerical values with confidence intervals**.
- Avoid vague statements like:
  - “significant clustering was observed”.

Better example:

Moran’s I indicated significant spatial autocorrelation (I = 0.34, p < 0.001).

**Conclusion**

Typical reviewer concern:

- Conclusions must **not exaggerate causal interpretation**.
- Avoid words like:
  - “determinants”
  - “cause”.

**3. Introduction**

**Line-by-Line Issues**

**Line ~35**

- The introduction lacks **global epidemiological context**.

You should cite:

- International Agency for Research on Cancer
- World Health Organization

**Line ~40**

The statement:

“Esophageal cancer is common in Ethiopia.”

This needs:

- **prevalence**
- **incidence**
- **mortality**

**Line ~45**

The manuscript should reference the **African esophageal cancer belt**.

Reviewer expectation:

- Discussion of high-risk areas from:
- African Esophageal Cancer Consortium

**Line ~50**

Research gap is **not clearly stated**.

You should answer:

- What spatial studies exist?
- What gap remains?

Example gap statement:

“However, little is known about the spatial distribution and clustering of esophageal cancer within Ethiopia.”

**4. Methods**

**Study Design**

**Line ~70**

- Cross-sectional design is mentioned but **time period is missing**.
- The reviewer must know:

Study period (e.g., 2015–2023)

**Study Area**

**Line ~80**

- Central Oromia must be defined:

Include:

- map
- administrative boundaries

Relevant entity:

- Oromia Region

**Data Source**

**Line ~90**

Issues:

- Case definition unclear.
- Were cases:

✔ Histologically confirmed
✔ clinically diagnosed

**Inclusion Criteria**

Missing information:

- age range
- residence duration
- duplicate records

**Geocoding**

**Line ~100**

The process of linking **kebele addresses to shapefiles** must be explained.

Questions:

- Was GPS used?
- Were coordinates approximated?

**Spatial Analysis**

**Line ~120**

Moran’s I should include explanation.

Expected formula:

I = spatial autocorrelation index

Reviewer suggestion:

Explain:

- Weight matrix
- Spatial neighbors

**Cluster Detection**

If **SaTScan** was used:

Please cite:

- SaTScan

And explain:

- scanning window
- maximum cluster size
- Monte Carlo simulations

**Gini Coefficient**

Reviewer concern:

Why is the **Gini coefficient used for spatial inequality**?

Needs justification.

**Ethical Approval**

Missing critical information:

- Institutional Review Board approval
- Data confidentiality

Expected example:

Ethical approval was obtained from Arsi University Institutional Review Board.

Relevant entity:

- Arsi University

**5. Results**

**Sample Description**

**Line ~150**

The manuscript should present:

- age distribution
- sex distribution
- residence distribution

A **table is required**.

**Spatial Autocorrelation**

Reviewer expects:

- Moran’s I value
- p-value
- interpretation

**Maps**

Spatial analysis requires **clear maps**:

Expected figures:

1️⃣ Study area map
2️⃣ Moran’s I cluster map
3️⃣ Hotspot map
4️⃣ Relative risk map

**Cluster Results**

For SaTScan clusters provide:

- cluster center
- radius
- relative risk
- p-value

**6. Discussion**

**Line ~210**

The discussion must begin with **key findings summary**.

Example:

This study identified significant spatial clustering of esophageal cancer in Central Oromia.

**Literature Comparison**

The manuscript currently lacks **comparison with African studies**.

Suggested comparisons:

- Kenya
- Tanzania
- Malawi
- Ethiopia previous studies

**Possible Explanations**

Reviewer expects discussion of:

- diet
- hot beverage consumption
- mycotoxin exposure
- tobacco
- alcohol

**Public Health Implications**

The discussion should include:

- screening programs
- cancer registry strengthening
- environmental risk assessment

**7. Limitations**

Important limitations missing:

1. hospital-based data
2. referral bias
3. missing exposure variables
4. geocoding inaccuracies

**8. Conclusion**

Conclusion should:

✔ summarize findings
✔ avoid speculation
✔ suggest policy action

**9. References**

Reviewer observations:

- Several citations appear **outdated**.
- Ensure **Vancouver format required by PLOS ONE**.

**Overall Reviewer Recommendation**

Major issues remain in:

1️⃣ Methods transparency
2️⃣ Spatial analysis explanation
3️⃣ Statistical reporting
4️⃣ Literature context

Recommendation:

**Major Revision Required**

**Reviewer name**: Yohannes Fekadu
