# Supplementary material for: Spatial clustering of esophageal cancer in central Oromia, Ethiopia: A spatial autocorrelation and scan statistics analysis
Source: PLoS One. 2026 Sep 15;21(9):e0357042. doi: 10.1371/journal.pone.0357042 (PMC13577588; doi:10.1371/journal.pone.0357042)
Supplement: Supplementary file 2 [file pone.0357042.s002.docx]

**General comment and overview**

The manuscript addresses an important public health issue geographical disparities in esophageal cancer in Ethiopia, using spatial analytical techniques. The topic is relevant and potentially impactful for policy and targeted interventions. However, several **methodological inconsistencies, reporting gaps, and clarity issues** need substantial revision before publication.

## **2. Title and Abstract**

### Strengths

- The title is clear and reflects the study design and scope and the abstract includes major sections (background, methods, results, conclusion).

### Major Concerns

**Abstract inconsistency and statistical errors**:

- The confidence interval for low-risk districts is incorrect: RR = 0.55; 95% CI: 1.81–2.10 (Page 2, lines 47–48). The CI contradicts the RR and must be corrected.
- Methods description is too dense and lacks clarity (Page 1–2, lines 31–39).
- The conclusion is vague and does not clearly reflect key quantitative findings.

### Recommendations

- Correct all statistical inconsistencies.
- Simplify and structure the methods section in the abstract.
- Include **clear quantitative highlights** (e.g., strongest cluster, magnitude of inequality).

## **3. Introduction / Background**

### Strengths

- Provides global and regional context with relevant statistics.
- Clearly identifies the research gap (lack of spatial modeling in Ethiopia).

### Major Issues

- **Excessive length and redundancy** (Pages 2–4, lines 60–106).
- Some statements lack proper linkage to study objectives.
- Overuse of global statistics without sufficient focus on local relevance.

### Recommendations

- Condense global burden discussion.
- Strengthen linkage between **background → gap → study objective**.
- Clearly justify why **Central Oromia** is uniquely important.

## **4. Research Questions and Hypotheses**

### Strengths

- Clearly stated research questions and null hypotheses (Page 5, lines 131–137).

### Issues

- Hypotheses are simplistic and do not fully reflect spatial modeling complexity.

### Recommendations

- Consider including **alternative hypotheses** and clarifying spatial assumptions.

## **5. Methods**

### 5.1 Study Design and Data Source

**Strengths**

- Multi-center data source improves coverage.

**Concerns**

- Cross-sectional design limits causal inference (Page 7, lines 184–188).
- Hospital-based data may introduce **selection bias** (referral bias not discussed).

**Recommendation**

- Explicitly acknowledge **selection bias and limited representativeness**.

### 5.2 Sampling and Sample Size

**Major Issues**

- Sample size calculation is **unclear and not aligned with spatial analysis** (Page 7–8, lines 197–210).
- Inclusion of all 367 cases contradicts initial sample size calculation logic.
- Use of **StatsToDo tool** is insufficiently justified scientifically.

**Recommendations**

- Clarify:
- Why Poisson-based sample size is appropriate.
- Why all cases were included despite calculated minimum.
- Consider reframing as **complete case inclusion** rather than sample-based study.

### 5.3 Data Collection and Measurement

**Strengths**

- Use of multiple data sources and trained collectors.

**Concerns**

- Verification of patient status via phone (Page 8, lines 226–227) raises:
- Ethical concerns (consent unclear)
- Potential recall bias
- High proportion of missing clinical variables (later seen in results).

**Recommendations**

- Clarify **consent procedures for follow-up contact**.
- Describe how missing data were handled.

### 5.4 Spatial Analysis Methods

**Strengths**

- Appropriate use of: Moran’s I, LISA, Gini coefficient, SaTScan

**Major Issues**

- **Formulas are unnecessarily detailed and disrupt readability** (Pages 9–10).
- No justification for: Choice of spatial weight matrix and 50% cluster size threshold (Page 11, line 305)
- Lack of explanation of: Model assumptions (Poisson model in SaTScan)
- Clearly describe **model assumptions and diagnostics**.

### 5.5 Data Quality Assurance

**Strengths**

- Includes training, pretesting, and validation.

**Concerns**

- “Face validity” and “content validity” lack methodological rigor (Page 12, lines 336–339).
- No reliability testing reported.

**Recommendation**

- Include **inter-rater reliability or consistency checks**.

## **6. Results**

### 6.1 Descriptive Findings

**Strengths**

- Comprehensive demographic and clinical description.

**Major Issues**

- Numerous **typographical errors**:
- “frmales” (Page 13, line 361)
- “Recidence” (Page 14)
- “Wieght loss” (Page 15)
- High proportion of missing data:
- Tumor differentiation (74%)
- Survival status (29%)

**Recommendation**

- Report missing data explicitly and discuss implications.

### 6.2 Statistical and Spatial Results

**Strengths**

- Multi-level spatial analysis (kebele, district, zone).
- Identification of hotspots and cold spots.

**Major Issues**

- **Inconsistent reporting**:
- Some results contradict abstract.
- Interpretation of Moran’s I:
- Woreda level non-significant but still discussed as meaningful (Page 18–19).
- Overinterpretation of LISA clusters without uncertainty discussion.

**Recommendations**

- Ensure consistency between abstract and results.
- Interpret non-significant findings cautiously.
- Include **maps with confidence or uncertainty indicators**.

## **7. Discussion (Implicit / Missing Section)**

### Major Gap

- The discussion section is **not fully presented** in the provided text but appears underdeveloped.

### Expected Improvements

- Compare findings with:
- East African esophageal cancer corridor literature.
- Discuss: Environmental and behavioral risk factors and Health system access issues

## **8. Ethical Considerations**

### Strengths

- Ethical approval clearly stated with protocol numbers.

### Concerns

Lack of clarity on:

Consent for patient follow-up contact

Data confidentiality procedures beyond storage

## **9. Data Availability**

### Major Issue

- Statement contradicts journal policy:

“Available upon request” is **not compliant with PLOS ONE policy**.

### Recommendation

- Deposit data in a **public repository** or provide justified restriction.
