# Supplementary material for: Spatial clustering of esophageal cancer in central Oromia, Ethiopia: A spatial autocorrelation and scan statistics analysis
Source: PLoS One. 2026 Sep 15;21(9):e0357042. doi: 10.1371/journal.pone.0357042 (PMC13577588; doi:10.1371/journal.pone.0357042)
Supplement: Supplementary file 3 [file pone.0357042.s004.docx]

**Response to reviewers:**

**We are grateful to the reviewers for addressing all sections of our manuscript. We are pleased that our manuscript was reviewed by experts and has been greatly enhanced. We have now revised the methods to improve the transparency of spatial analysis and statistical reporting. Additionally, we have carefully revised the literature section according to the reviewers’ request. In general, we have revised the entire manuscript as requested by the journal's editor and the two reviewers. Our responses to both reviewers are given for each comment one by one. If more clarifications are needed, we are happy to provide them upon request.**

**Reviewer #1:**

1. **Title
   Line 1: The title is clear but could be improved by indicating the analytical methods (e.g., spatial scan statistics, Moran’s I). Consider specifying study period if applicable. Suggested revision example:**

**Response:** Thank you very much for this comment. The comment is well taken, and the title has been revised as “Spatial Clustering of Esophageal Cancer in Central Oromia, Ethiopia: A Spatial Autocorrelation and Scan Statistics analysis (line 1)”.

2. **Abstract**
   2.1. **Background, Line 6–9, the sentence structure is unclear and grammatically inconsistent. “GLOBOCAN report showed” should be written as “According to the GLOBOCAN 2022 report”. The statement “with the two zones having the highest prevalence (68.3%)” needs a clear citation or explanation of the calculation method. “Undertaking thorough study” should be “a thorough study”.**

**Response:** Thank you very much for the comment. We have revised the background section accordingly. For the statement, ‘with the two zones (Arsi and Bale) having the highest prevalence (68.3%) and mortality,’ we cited Reference #20 in the introduction section of the main manuscript (Lines 91-93). However, according to the PLOS ONE journal guidelines, references are not permitted in the abstract section.”

2.2. **Reviewer concern: The background mixes global statistics and regional statistics without clear transition.**

**Response:** Thank you very much for the feedback. We have revised the introduction section of the abstract accordingly (Lines 17-23).

2.3. Objective, Line 12; the objective is clear but too broad, Consider specifying methodological approach. Example improvement: “To assess spatial clustering and identify geographic hotspots of esophageal cancer in Central Oromia using spatial autocorrelation and spatial scan statistics.”

**Response:** Thank you very much for the feedback. Since the method of analysis, that is, spatial autocorrelation and spatial Scan statistics, is now included in the title (line 1) and well explained in the methods section, particularly in the data analysis section (lines 306-342), we prefer to keep the previous version of the objective. Additionally, spatial clustering includes spatial inequality, which was measured by the Gini coefficient, spatial autocorrelation, which was measured by Moran's I and Scan Statistics which was focused on measuring the probability of developing esophageal cancer.

2.4. Methods, Line 15–21

Issues:

2.4.1. Study design; “The cross-sectional study design was employed” should specify retrospective record review.

**Response:** Thank you very much for the feedback. It is included accordingly (lines 27-30).

2.4.1. Sample size; “367 patients collected from records” should be clarified: What years were included? Were cases confirmed histologically?

**Response:** Thank you very much for this important observation. Some revisions have been made accordingly. The sample size calculation procedure followed the assumptions of the Poisson distribution, as esophageal cancer is a rare disease. We calculated the sample size to determine whether the total number of available patients in the selected hospitals was sufficient to conduct this research (detail, lines 193-211). In addition, we have a subsequent follow-up study planned based on the findings of the current study. The follow-up study will recruit participants from those included in this study; therefore, we included all patients who visited the selected hospitals **between April 3, 2019, and August 25, 2025 (line 30)**. As it is explained under source population and eligibility section of the materials and method, eligible participants were adults (≥18 years) from the five zones with an endoscopic and/or histological confirmation of esophageal cancer who attended the study hospitals (lines 184-192).

2.4.2. Hospital names, Ensure correct spelling: “Tikur Anmbassa” → Tikur Anbessa

**Response:** Thank you very much for the feedback. It is edited accordingly and used consistently throughout the manuscript (line 28).

2.4.3. Geographic variables, you mention kebele, woreda, and zone, but it is unclear which level was used in the analysis?

**Response**: **Thank you very much for the feedback. We considered all levels during analysis, as shown in the results section. The detailed analysis approach is explained in the data analysis section and supported by Fig. 3 (lines 311-312). However, because of PLOS ONE's word count limitations for abstracts, we only included a summary in the abstract.**

2.4.4. Spatial data source, The Ethiopian Space Science and Geospatial Institute should be cited properly: Ethiopian Space Science and Geospatial Institute.

**Response: Thank you very much for the feedback. We received the raw data of the Ethiopian shapefile from the Ethiopian Space Science and Geospatial Institute, which includes region, zone, district, and kebele-level layers. We then linked this shapefile to the data collected from the four hospitals using the patients' kebele of residence. We have a letter from the institute confirming that we received the data and granting permission to use it for publication. We can upload this letter as a supporting file to the journal's portal. Otherwise, we have no reference to cite.**

2.4.5. Statistical methods, the abstract mentions Gini coefficient, Moran’s I, Relative risk and Log likelihood ratio. Reviewer concern: The software used (SaTScan, ArcGIS, R) should be mentioned.

**Response: Thank you for this important feedback. We used SaTScan version 10.3.3 for cluster identification and estimation of the magnitude of risks within the clusters; GeoDa 1.22.01.21 to identify spatial autocorrelation and determine neighborhood relationships; and Quantum Geographic Information System (QGIS) desktop version 3.12 for spatial description and visualization. In addition, the data were imported into R software version 4.3.3 for further descriptive analysis (Choropleth maps), inferential analysis, and spatial inequality assessment. These software are explained in the data processing and management section of the manuscript (lines 298-305). However, because of PLOS ONE's word count limitations for abstracts, we only included key methods in the abstract. If the journal's editorial board allows, we are happy to add more detail.**

2.5. Results, Common issues observed in the manuscript style:
     2.5.1. Results must include numerical values with confidence intervals.

**Response:** Thank you for the feedback. Yes, we agreed with you and did the same thing.

2.5.2. Avoid vague statements like: “significant clustering was observed”. Better example: Moran’s I indicated significant spatial autocorrelation (I = 0.34, p < 0.001).

**Response:** Thank you very much for the comment and elaboration. It is revised accordingly (lines 38-41).

2.6. Conclusion, Typical reviewer concern: Conclusions must not exaggerate causal interpretation. Avoid words like “determinants” and “cause”.

**Response:** Thank you very much for the concern. We did not include such wording in the conclusion because our study focused on the identification of spatial clusters and the probability of developing the disease within those clusters.

3. Introduction,
   3.1. Line ~35, the introduction lacks global epidemiological context. You should cite International Agency for Research on Cancer and World Health Organization

**Response: Thank you for the valuable comment. The introduction section of our manuscript spans lines 59-130. In the revised manuscript, we addressed the global burden of esophageal cancer in lines 74-82. Additionally, we have cited references from IARC and WHO on esophageal cancer, for example, in paragraph one (lines 59–64, refs. 1–4) and paragraph five (line 85, ref. #18). Moreover, we are happy to add more upon your recommendation of any specific references we may have missed.**

3.2. Line ~40, the statement “Esophageal cancer is common in Ethiopia.” This needs, prevalence, incidence and mortality

**Response:** **Thank you very much for the comment. Your comment is well taken. We addressed the burden of esophageal cancer in Ethiopia in lines 83–93.**

3.3. Line ~45; the manuscript should reference the African esophageal cancer belt. Reviewer expectation is Discussion of high-risk areas from African Esophageal Cancer Consortium

**Response:** Thank you very much for this comment. Your comment is well accepted. We wrote about this in lines 96-10.

3.4. Line ~50, Research gap is not clearly stated. You should answer, what spatial studies exist? What gap remains? Example gap statement: “However, little is known about the spatial distribution and clustering of esophageal cancer within Ethiopia.”

**Response: Thank you very much for the comment and elaboration. We accepted the comment and revised the gaps (lines 109–120).**

4. Methods
   4.1. Study Design, Line ~70, Cross-sectional design is mentioned but time period is missing. The reviewer must know: Study period (e.g., 2015–2023)

**Response: Thank you very much for the feedback. We have already included the study period under the study design and period section as “Data collection took place from December 15, 2024, to August 25, 2025” (line 183). Additionally, we included in the manuscript that the study included patients who visited the four hospitals between April 3, 2019, and August 25, 2025, under the source population and eligibility section (line 189).**

4.2. Study Area, Line ~80, Central Oromia must be defined. Include, map, administrative boundaries, Relevant entity: Oromia Region

**Response: Thank you very much for this comment. Your point is well taken. However, we have already defined Central Oromia as including five adjoining zones, namely Arsi, West Arsi, Bale, East Bale, and East Shewa. Additionally, we have explained the boundaries of each zone and illustrated this with a map (lines 134-170, Fig 1).**

4.3. Data Source, Line ~90, Case definition unclear. Were cases, Histologically confirmed and clinically diagnosed

**Response:** **Thank you for raising this important comment. Your point is well taken. We have clearly defined case confirmation diagnosis in the manuscript under the source population and eligibility criteria section as follows: “Eligible participants were adults (≥18 years) from these zones with endoscopic and/or histological confirmation of esophageal cancer who attended the study hospitals between April 3, 2019, and August 25, 2025” (lines 185–192).**

4.4. Inclusion Criteria, Missing information: age range, residence duration and duplicate records

**Response: Thank you very much for this important comment. As we have noted under the source population and eligibility section, we included all patients aged 18 years and above for whom the kebele of residence was recorded. Since the duration of residence was not recorded for all patients, we did not consider duration as an inclusion criterion. We excluded duplicate records using the patient's card number (lines 189-192).**

4.5. Geocoding, Line ~100, the process of linking kebele addresses to shapefiles must be explained. Was GPS used? Were coordinates approximated?

**Response:** Thank you for this important comment. We have clarified the geocoding process in the manuscript (lines 227-232). GPS devices were not used for data collection. Instead, coordinates were approximated by assigning each patient the geometric centroid of their recorded kebele, derived from the Ethiopian shapefile obtained from the Ethiopian Space Science and Geospatial Institute. This approach is standard for kebele-level spatial analysis when exact GPS coordinates are not available. Patients without recorded kebele were excluded from spatial analysis

4.6. Spatial Analysis, Line ~120, Moran’s I should include explanation. Expected formula: I = spatial autocorrelation index. Reviewer suggestion: Explain: Weight matrix and Spatial neighbors

Response: **Thank you very much for this important feedback. In our manuscript, particularly in the 'operational definitions and measurements' section, we explained Moran's I in detail, including its formula (lines 274-282). We also explained the construction of the spatial weight matrix using queen contiguity and defined spatial neighbors as kebeles sharing common boundaries (lines 283-286).**

4.7. Cluster Detection. If SaTScan was used, Please cite, SaTScan and explain: scanning window, maximum cluster size and Monte Carlo simulations,

**Response: Thank you very much for this important comment. We have now cited the reference for SaTScan in the manuscript accordingly (lines 299-301). Under the data analysis section, we included an explanation of the circular scanning window, specified that the maximum cluster size was set to 50% of the population at risk, and provided a description of Monte Carlo simulations (lines 312-316).**

4.8. Gini Coefficient, Reviewer concern: Why is the Gini coefficient used for spatial inequality? Needs justification.

**Response:** Thank you for this important comment. We have now added a justification for using the Gini coefficient in the manuscript, under ‘Operational definitions and measurements’ section (lines 270-273). Specifically, we explained that The Gini coefficient provides a standardized, easily interpretable index that summarizes the degree of inequality in disease burden across geographic areas. The Gini coefficient is less sensitive to outliers and allows for comparison with other studies of spatial inequality in esophageal cancer outcomes.

4.9. Ethical Approval, Missing critical information: Institutional Review Board approval and Data confidentiality. Expected example: Ethical approval was obtained from Arsi University Institutional Review Board. Relevant entity: Arsi University

**Response: Thank you very much for this important comment and elaboration. We have included this under the 'Ethics approval and consent to participate' section (lines 394-396) as follows: 'Ethical approval was obtained from the Institutional Review Board (IRB) of the College of Health Sciences, Addis Ababa University (Protocol No. 088/24/SPH), as well as from the IRB of the Oromia Regional State Health Bureau (Protocol No. BFO/H2/032/17)'. Additionally, we have attached both letters to the PLOS ONE portal as supporting letters.**

5. **Results**
   5.1. Sample Description, Line ~150, the manuscript should present, age distribution, sex distribution, residence distribution and a table is required.

**Response: Thank you very much for this comment. Yes, you are right, and we did the same thing under the 'Socio-demographic characteristics of esophageal cancer patients' section. We also supported this part with Table 1 (lines 409-421).**

5.2. Spatial Autocorrelation, Reviewer expects, Moran’s I value, p-value and interpretation

**Response: Thank you very much for this comment. Yes, you are right, and we did the same thing under the ‘**Spatial autocorrelation of esophageal cancer’ **section. We also supported it with interpretation and Table 4 (lines 486-512).**

5.3. Maps, Spatial analysis requires clear maps: Expected figures: Study area map, Moran’s I cluster map, Hotspot map, and Relative risk map

**Response:** **Thank you very much for this comment. We attached the figures independently as separate figure files. Figure one shows the study area map (Fig. 1), and Figure seven shows the Moran's I cluster map and hotspot map (Fig. 7). For relative risk map, we included the maps showing the location of clusters and their relative risk (RR) as supporting files (S1_File to S4_File) (lines 810-818). If further clarification is required we are happy to provide it.**

5.4. Cluster Results, For SaTScan clusters provide: cluster center, radius, relative risk, p-value

Response: Thank you very much for this important comment. Your comment is considered, and we have revised the SaTScan cluster result section accordingly (lines 520-539).

6. Discussion
   6.1. Line ~210, the discussion must begin with key findings summary. Example: This study identified significant spatial clustering of esophageal cancer in Central Oromia.

**Response: Thank you very much for the important comment. Your comment is well taken. The first paragraph of our manuscript's discussion section has been revised accordingly (lines 562-569).**

6.2. Literature Comparison, The manuscript currently lacks comparison with African studies. Suggested comparisons: Kenya, Tanzania, Malawi and Ethiopia previous studies

**Response: Thank you very much for this important comment. We could not identify studies conducted using the same methodology to allow for detailed comparison. However, we attempted to discuss geographic variation in esophageal cancer occurrence in relation to variations in potential risk factors reported within the East African esophageal cancer corridor literature. In addition, we incorporated findings from other studies conducted in the region, including studies from Kenya, Tanzania, Malawi, and Ethiopia (lines 604-615).**

6.3. Possible Explanations, Reviewer expects discussion of diet, hot beverage consumption, mycotoxin exposure, tobacco and alcohol

**Response:** **Thank you very much for this comment. Your comment is well taken. However, the primary focus of this study was the spatial clustering of esophageal cancer, on which our discussion section is centered. Accordingly, in the discussion section, we attempted to relate the observed clustering patterns to the identified and putative risk factors reported in the East African esophageal cancer literature, including hot beverages, lack of fruits and vegetables, behavioral risk factors, poor oral health, and environmental carcinogens such as polycyclic aromatic hydrocarbons (PAHs) (lines 604-606).**

6.4. Public Health Implications; The discussion should include, screening programs, cancer registry strengthening, and environmental risk assessment

**Response:** Thank you very much for this important comment. This was discussed in lines 607-615.

7. Limitations, Important limitations missing: hospital-based data, referral bias, missing exposure variables, geocoding inaccuracies

**Response:** Thank you for this important comment. We agree that these limitations should be acknowledged. Accordingly, we have revised the limitations section to include the use of hospital-based data and potential selection bias; referral bias; absence of individual-level exposure variables; and geocoding inaccuracies due to the use of kebele centroids (lines 616-626).

8. Conclusion, Conclusion should: summarize findings, avoid speculation and suggest policy action

**Response:** Thank you very much for this important comment. Your comment is well taken, and we have revised the conclusion section accordingly (lines 67-637).

9. References, Reviewer observations: Several citations appear outdated. Ensure Vancouver format required by PLOS ONE.

**Response:** **Thank you very much for this comment. Yes, you are right. We used a few older references that contain very important information on the Gini coefficient, Moran's I, and scan statistics. Otherwise, we followed the PLOS ONE guidelines for reference formatting. Moreover, we are happy to make revisions if you recommend any specific references to add or replace.**

**Reviewer #2:**

1. **General comment and overview:** The manuscript addresses an important public health issue geographical disparities in esophageal cancer in Ethiopia, using spatial analytical techniques. The topic is relevant and potentially impactful for policy and targeted interventions. However, several **methodological inconsistencies, reporting gaps, and clarity issues** need substantial revision before publication.

**Response:** Thank you very much for your encouragement and kind words. We have revised the entire manuscript in accordance with the journal’s guidelines, as well as the editors’ and reviewers’ comments. We have addressed each comment one by one.

2. **Title and Abstract**

### Major Concerns

### **Abstract inconsistency and statistical errors**:

2.1. **The confidence interval for low-risk districts is incorrect: RR = 0.55; 95% CI: 1.81–2.10 (Page 2, lines 47–48). The CI contradicts the RR and must be corrected.**

**Response:** Thank you very much for the comment; it is an excellent observation. It was a typing error and has been corrected as follows: (RR = 0.55; 95% CI: 0.41, 0.73) (line 45).

2.2. **Methods description is too dense and lacks clarity (Page 1–2, lines 31–39).**

**Response:** **Thank you very much for the comment. We have simplified and restructured the texts. We are happy to address the comment as required (lines 26-36).**

2.3. T**he conclusion is vague and does not clearly reflect key quantitative findings.**

### Response: **Thank you very much for this comment as well. We have clarified and added some key quantitative findings (lines 48-56).**

### 3. ****Introduction / Background****

### Major Issues

3.1. **Excessive length and redundancy** (Pages 2–4, lines 60–106).

**Response:** **Thank you for the comment. Your point is well taken, and we have revised the text accordingly. This section is now well condensed, and redundancies have been removed (lines 59-120).**

3.2. Some statements lack proper linkage to study objectives. Strengthen linkage between **background → gap → study objective**.

**Response:** Thanks a lot for the comment. It is excellent observation. Now we revised it and kept those linked to our objective (Lines 59-120).

3.3. Overuse of global statistics without sufficient focus on local relevance. Condense global burden discussion. Clearly justify why **Central Oromia** is uniquely important.

**Response:** Thank you very much for this important comment. your comment is well taken and we have revised the introduction part accordingly. The global statistics of the esophageal cancer is explained in the lines 74-86. Central Oromia, as defined in this study, includes five zones; Arsi, West Arsi, Bale, East Bale, and East Showa (lines 134-170). Reports have shown that esophageal cancer is highly prevalent in this study area. However, no study to date has investigated the disease clustering in the area, which has the potential to identify the populations at risk. This is why the study focused on this area. We revised the introduction accordingly (lines 106-115).

4. **Research Questions and Hypotheses**

### Issues

4.1. Hypotheses are simplistic and do not fully reflect spatial modeling complexity. Consider including **alternative hypotheses** and clarifying spatial assumptions.

**Response:** Thanks you very much for the valuable comment. The alternative hypotheses have been included in the revised manuscript as you suggested (Lines 125-131). The model assumptions are explicitly explained in the Method and Materials section (lines 318-328).

5. **Methods**

## 5.1. **Study Design and Data Source**

**Concerns**: Cross-sectional design limits causal inference (Page 7, lines 184–188). Hospital-based data may introduce **selection bias** (referral bias not discussed). Explicitly acknowledge **selection bias and limited representativeness**.

**Response:** Thank you very much for this valuable comment. Yes, it is know that cross-sectional study design has an ambiguity when investigating causal pathway. However, the main focus of this study was to investigate the existence of spatial clusters using Gini coefficient, autocorrelation by Moran’s I and the probability of developing esophageal cancer within the clusters. We have revised the manuscript and acknowledged these limitations in this section (lines 181-183) as well as at the end of the discussion section (lines 616-626).

### 5.2. Sampling and Sample Size

**Major Issues**

5.2.1. **Sample size calculation is unclear and not aligned with spatial analysis (Page 7–8, lines 197–210). Inclusion of all 367 cases contradicts initial sample size calculation logic.**

**Response:** Thank you very much for this important observation. Some revisions have been made accordingly. The sample size calculation procedure followed the assumptions of the Poisson distribution, as esophageal cancer is a rare disease. We calculated the sample size to determine whether the total number of available patients in the selected hospitals was sufficient to conduct this research.

In addition, we have a subsequent follow-up study planned based on the findings of the current study. The follow-up study will recruit participants from those included in this study; therefore, we included all patients who visited the selected hospitals during the study period (lines 193-221).

If the inclusion of the sample size calculation is out of the scope of the journal’s policy, we would be happy to remove it.

5.2.2. **Use of** **StatsToDo tool** **is insufficiently justified scientifically.**

**Response:** Thank you for this important inquiry. We have made the necessary amendments accordingly. StatsToDo is a free online statistical platform that provides calculators for a range of statistical analyses and research methods, including sample size calculation, power analysis, statistical testing, data analysis support, and epidemiological and biomedical research calculations. The specific tool we used is designed to estimate sample size for Poisson-distributed count data, which is appropriate for rare events or diseases such as esophageal cancer. We have also added the reference in the relevant section to provide further information about the software and its functionality (lines 193-221).

### 5.3. Data Collection and Measurement

5.3.1. **Concerns**: Verification of patient status via phone (Page 8, lines 226–227) raises: Ethical concerns (consent unclear), and potential recall bias: Clarify **consent procedures for follow-up contact.**

**Response:** Thank you very much for your excellent observation. We revised and amended the manuscript accordingly. We contacted patients whose health outcome status (i.e., survival status) was not recorded but for whom tracing mechanisms were available, as this information was an important input for our subsequent follow-up survival study. This process was conducted after obtaining ethical clearance from the regional IRB and the Addis Ababa University College of Health Sciences IRB, as described in the ethical clearance section (lines 393-407). Additionally, verbal consent was obtained from all contacted patients who were alive at the time of communication. Regarding recall bias, only information on patients’ health outcomes, specifically whether they alive or deceased at the time of data collection, was obtained through direct communication. All other study variables were extracted from patients’ medical records. Therefor the likelihood of recall bias was minimal (lines 222-224).

5.3.2. **High proportion of missing clinical variables (later seen in results). Describe how missing data were handled.**

**Response:** Thank you very much for raising this important comment. As clearly stated in the abstract, the primary objective of the study was to identify the spatial clustering of esophageal cancer cases. This analysis was conducted using the Gini coefficient, Moran’s I, and SaTScan at the Kebele and Woreda/District levels. As described in the source population and eligibility criteria sections, we included all patients whose Kebele information was recorded (lines 189-192). Therefore, the incompleteness of some socio-demographic and clinical variables had minimal impact on the main objective of the study. However, for transparency and descriptive purposes, we explicitly reported the extent of missing information.

### 5.4. Spatial Analysis Methods

**Major Issues**

5.4.1. **Formulas are unnecessarily detailed and disrupt readability** (Pages 9–10).

**Response:** Thank you very much for the comment. We included these formulas to demonstrate how we measured the study objectives and performed the analysis for the identification of esophageal cancer clusters. As we were unable to find a similar study, we provided a detailed explanation to enhance the reproducibility of the methods in future research (lines 234-282). However, we can reduce the level of detail and remove the formulas upon your recommendation.

5.4.2. **No justification for: Choice of spatial weight matrix and 50% cluster size threshold (Page 11, line 305)**

**Response: Thank you very much for this important comment. We have revised the statement as follows: “The upper spatial cluster size was set to 50% of the total population at risk, as recommended by Kulldorff (2018), to enhance comparability across detected clusters. Statistical significance was assessed using 999 Monte Carlo replications.” Additionally, we have cited the reference. We chose this approach because using a percentage of the population at risk avoids the misleading identification of clusters that can occur with a fixed radius, for example, short radii capture dense urban populations while long radii capture sparse rural populations, making comparisons difficult (lines 311-316).**

5.4.3. **Lack of explanation of: Model assumptions (Poisson model in SaTScan), Clearly describe model assumptions and diagnostics.**

**Response:** Thanks a lot for this valuable comment. We added the model assumption test result in the data analysis section as follows: **“**The discrete Poisson model was used to determine the magnitude of risk within the clusters and was interpreted using the risk ratio and its 95% confidence interval. Assessment of the Poisson distribution assumption at the Kebele level demonstrated a dispersion index of 1.06, indicating that the variance was approximately equal to the mean (mean = 1.51; variance = 1.61). Furthermore, the over-dispersion test was not statistically significant (p = 0.4409), providing no evidence of substantial over-dispersion and supporting the suitability of the Poisson model for the spatial analysis (lines 318-325)”.

### 5.5. Data Quality Assurance

**Concerns**

5.5.1. “Face validity” and “content validity” lack methodological rigor (Page 12, lines 336–339).

**Response:** **Thank you very much for the important comment. We revised the section accordingly as follows: "Furthermore, the content and face validity of the data extraction form were evaluated prior to data collection. Content validity was assessed through an extensive review of relevant literature and expert evaluation by two specialists with experience in oncology and epidemiological research to ensure the relevance and comprehensiveness of the tool. Face validity was assessed by examining the feasibility, readability, consistency of formatting, and clarity of the language used in the data extraction form. Necessary revisions were made based on the feedback obtained" (lines 374–379).**

5.5.2. No reliability testing reported. Include **inter-rater reliability or consistency checks**.

**Response:** Thank you very much for this valuable comment. We revised this section to better describe the procedures performed, as follows: ‘Prior to the actual data collection, the tool was pretested on 10 (5%) esophageal cancer patient records from the selected hospitals to identify potential issues related to wording, interpretation, and compatibility with hospital record formats. Inter-rater reliability was assessed by having four independent data collectors review the same 10 records. Fleiss’ Kappa was used to assess agreement for categorical variables, including sex, zone, residence, religion, type of diagnosis, diagnostic methods used, cancer stage, types of treatment provided, patients’ survival status, signs and symptoms, degree of tumor differentiation, tumor location, and histopathological subtype.

The Intra-class Correlation Coefficient (ICC) was used to evaluate agreement for age of the patients. Accordingly, the pretest demonstrated substantial to almost perfect agreement among the four raters. Fleiss’ Kappa values were 0.78 for sex (p < 0.001), 0.70 for zone (p < 0.001), 0.73 for residence (p < 0.001), 0.92 for religion (p < 0.001), 0.75 for type of patient diagnosed (p < 0.001), 0.89 for diagnostic methods used (p < 0.001), 0.91 for cancer stage (p < 0.001), 0.86 for type of treatment provided (p = 0.001), 0.77 for patients’ survival status (p < 0.001), 0.92 for presenting signs and symptoms (p < 0.001), 0.87 for degree of tumor differentiation (p < 0.001), 0.91 for tumor location (p < 0.001), and 0.91 for histopathological subtype (p < 0.001).

The Intraclass Correlation Coefficient (ICC) for age was 0.99 (95% CI: 0.971–0.997; p < 0.001), indicating excellent consistency among the raters. The combined assessment of all categorical variables showed almost perfect agreement among the four abstractors, with a Fleiss’ Kappa value of 0.92 (p < 0.001). All discrepancies identified during the pretest were discussed and resolved, and the data abstraction tool was refined accordingly prior to the actual data collection (lines 351-379).’

## 6. **Results**

## 6.1. **Descriptive Findings**

**Major Issues**

6.1.1. Numerous **typographical errors**: “frmales” (Page 13, line 361), “Recidence” (Page 14), “Wieght loss” (Page 15)

**Response:** Thank you very much for the comment. We apologize for the typographical errors. The entire manuscript has now been carefully revised and edited.

6.1.2. High proportion of missing data: Report missing data explicitly and discuss implications. Tumor differentiation (74%), Survival status (29%)

**Response:** Thank you very much again for this important comment. We have addressed this issue under Section “5.3.2.” as follows: ‘As clearly stated in the abstract, the primary objective of the study was to identify the spatial clustering of esophageal cancer cases. This analysis was conducted using the Gini coefficient, Moran’s I, and SaTScan at the Kebele and Woreda/District levels. As described in the source population and eligibility criteria sections, we included all patients whose Kebele information was recorded. Therefore, the incompleteness of some socio-demographic and clinical variables had minimal impact on the main objective of the study. However, for transparency and descriptive purposes, we explicitly reported the extent of missing information (lines 189-192).’

### 6.2.  Statistical and Spatial Results

**Major Issues**

6.2.1. **Inconsistent reporting**: Some results contradict abstract. Recommendation: Ensure consistency between abstract and results.

**Response:** Thank you very much for this comment. We made minor amendments to the abstract accordingly. However, the spatial analysis identified three main findings. The first was spatial inequality, measured using the Gini coefficient; the second was spatial autocorrelation, assessed using Global Moran’s I and Local Moran’s I (LISA); and the third was the probability of developing esophageal cancer, identified using SaTScan and measured by the relative risk (RR), along with its 95% confidence interval and p-value. Therefore, we attempted to summarize these key findings in the abstract as well (lines 37-47). We would be happy to make further revisions if you could specify which result appears to contradict the abstract.

6.2.2. Interpretation of Moran’s I: Woreda level non-significant but still discussed as meaningful (Page 18–19). Over-interpretation of LISA clusters without uncertainty discussion. **Recommendations**: Interpret non-significant findings cautiously. Include **maps with confidence or uncertainty indicators**.

**Response:** Thank you very much for this comment. We removed the non-significant finding of wored from the discussion section. However, for transparency purposes, we retained it in the descriptive results section. Regarding the interpretation of the LISA analysis, because its nature is to provide detailed information for each Woreda and Kebele, we reported the actual findings obtained from the analysis (lines 486-510). We would be happy to further reduce or revise any findings that may appear over-interpreted upon your recommendation.

## 7. **Discussion (Implicit / Missing Section)**

### Major Gap

7.1. **The discussion section is not fully presented in the provided text but appears underdeveloped. Compare findings with: East African esophageal cancer corridor literature.**

**Response:** Thank you very much for this comment. We could not identify studies conducted using the same methodology to allow a detailed comparison. However, we attempted to discuss the geographic variation in esophageal cancer occurrence in relation to variations in potential risk factors reported within the East African esophageal cancer corridor literature. In addition, we incorporated findings from other studies conducted in the region (lines 604-615).

7.2. **Discuss: Environmental and behavioral risk factors and Health system access issues**

**Response:** Thank you very much for this comment. The comment is well taken. However, the primary focus of this study was the spatial clustering of esophageal cancer in which our discussion section focused. Accordingly, in the discussion section, we attempted to relate the observed clustering patterns to the identified and putative risk factors reported in the East African esophageal cancer literature including hot beverages, lack of fruits and vegetables, behavioral risk factors, poor oral health, and environmental carcinogens such as polycyclic aromatic hydrocarbons (PAHs) (lines 604-615).

## 8. **Ethical Considerations**

### Concerns: Lack of clarity on Consent for patient follow-up contact and Data confidentiality procedures beyond storage

### Response: Thank you very much for this important comment. We revised the ethical considerations section to clarify the consent process for patient follow-up contact and the procedures used to maintain data confidentiality. Verbal consent was obtained from patients contacted through the tracing mechanism prior to data collection. In addition, patient identifiers were removed from the dataset, unique codes were assigned, and access to the data was restricted to the research team. All electronic data were password protected and reported anonymously (lines 394-407).

## 9. **Data Availability**

### Major Issue: Statement contradicts journal policy; “Available upon request” is **not compliant with PLOS ONE policy**. Recommendation: Deposit data in a **public repository** or provide justified restriction.

### Response: Thank you for this comment. This section has been revised as follows: ‘The data underlying this study have been deposited in the repository of Addis Ababa University. Additionally, the data can be uploaded to the PLOS ONE journal portal as a supporting file upon acceptance (lines 660-665).’

~The End~
