# Supplementary material for: Spatial clustering of esophageal cancer in central Oromia, Ethiopia: A spatial autocorrelation and scan statistics analysis
Source: PLoS One. 2026 Sep 15;21(9):e0357042. doi: 10.1371/journal.pone.0357042 (PMC13577588; doi:10.1371/journal.pone.0357042)
Supplement: Peer review history

# Peer review: Decision Letter 0

**DOI:** 10.1371/journal.pone.0357042.r001

**Authors:** Takele Gezahegn Demie

17 Apr 2026

Dear Dr. Beyen,

Thank you for submitting your manuscript to PLOS ONE. After careful consideration, we feel that it has merit but does not fully meet PLOS ONE’s publication criteria as it currently stands. Therefore, we invite you to submit a revised version of the manuscript that addresses the points raised during the review process.

The manuscript addresses an important and underexplored public health issue, examining spatial disparities in esophageal cancer in Ethiopia. Both reviewers agree that the topic is relevant and potentially impactful for policy and targeted interventions. However, significant concerns remain across multiple sections of the manuscript.

**The revisions should focus on:**

- **Title and Abstract** : Ensure consistency, correct statistical reporting, and highlight key quantitative findings without exaggeration.
- **Introduction** : Condense background material, strengthen the link between global context and local relevance, and clearly articulate the research gap and objectives.
- **Methods** : Improve transparency in study design, sampling, and spatial analysis procedures. Justify methodological choices, acknowledge limitations such as hospital-based data and selection bias, and clarify assumptions.
- **Results** : Correct inconsistencies, report-missing data explicitly, ensure alignment with the abstract, and interpret findings cautiously with appropriate confidence intervals and uncertainty measures.
- **Discussion** : Expand and strengthen by comparing findings with regional and international literature, exploring plausible risk factors, and situating results within broader public health implications.
- **Ethics and Data Availability** : Clarify consent and confidentiality procedures, and revise the data availability statement to comply with journal policy.
- **Presentation** : Address typographical errors, improve grammar and phrasing, and standardize tables, references, and figures (including maps).

Detailed comments from our esteemed reviewers are also attached or appended below.

Please submit your revised manuscript by Jun 01 2026 11:59PM. If you will need more time than this to complete your revisions, please reply to this message or contact the journal office at plosone@plos.org. When you're ready to submit your revision, log on to https://www.editorialmanager.com/pone/ and select the 'Submissions Needing Revision' folder to locate your manuscript file.

Please include the following items when submitting your revised manuscript:

- A letter that responds to each point raised by the academic editor and reviewer(s). You should upload this letter as a separate file labeled 'Response to Reviewers'.
- A marked-up copy of your manuscript that highlights changes made to the original version. You should upload this as a separate file labeled 'Revised Manuscript with Track Changes'.
- An unmarked version of your revised paper without tracked changes. You should upload this as a separate file labeled 'Manuscript'.

If you would like to make changes to your financial disclosure, please include your updated statement in your cover letter. Guidelines for resubmitting your figure files are available below the reviewer comments at the end of this letter.

If applicable, we recommend that you deposit your laboratory protocols in protocols.io to enhance the reproducibility of your results. Protocols.io assigns your protocol its own identifier (DOI) so that it can be cited independently in the future. For instructions see: https://journals.plos.org/plosone/s/submission-guidelines#loc-laboratory-protocols. Additionally, PLOS ONE offers an option for publishing peer-reviewed Lab Protocol articles, which describe protocols hosted on protocols.io. Read more information on sharing protocols at https://plos.org/protocols?utm_medium=editorial-email&utm_source=authorletters&utm_campaign=protocols.

As the corresponding author, your ORCID iD is verified in the submission system and will appear in the published article. PLOS supports the use of ORCID, and we encourage all coauthors to register for an ORCID iD and use it as well. Please encourage your coauthors to verify their ORCID iD within the submission system before final acceptance, as unverified ORCID iDs will not appear in the published article. *Only*  the individual author can complete the verification step; PLOS staff *cannot*  verify ORCID iDs on behalf of authors.

We look forward to receiving your revised manuscript.

Kind regards,

Takele Gezahegn Demie, MPH

Academic Editor

PLOS One

**Journal Requirements:**

When submitting your revision, we need you to address these additional requirements.

1. Please ensure that your manuscript meets PLOS ONE's style requirements, including those for file naming. The PLOS ONE style templates can be found at

https://journals.plos.org/plosone/s/file?id=wjVg/PLOSOne_formatting_sample_main_body.pdf and

https://journals.plos.org/plosone/s/file?id=ba62/PLOSOne_formatting_sample_title_authors_affiliations.pdf

2. We note that the grant information you provided in the ‘Funding Information’ and ‘Financial Disclosure’ sections do not match.

When you resubmit, please ensure that you provide the correct grant numbers for the awards you received for your study in the ‘Funding Information’ section.

3. Thank you for stating the following financial disclosure:

“The research was funded by the DELTAS Africa II programme through the Research and Excellence in African Capacity to Control and Treat Cancer (REACCT-CAN) and Network for Oncology Research in Sub-Saharan Africa (NORA) consortiums, which aim to strengthen cancer research capacity across Africa by supporting multidisciplinary research, training, and collaboration to improve cancer prevention, early detection, treatment, and care.”

Please state what role the funders took in the study.  If the funders had no role, please state: "The funders had no role in study design, data collection and analysis, decision to publish, or preparation of the manuscript."

If this statement is not correct you must amend it as needed.

Please include this amended Role of Funder statement in your cover letter; we will change the online submission form on your behalf.

4. Please note that funding information should not appear in any section or other areas of your manuscript. We will only publish funding information present in the Funding Statement section of the online submission form. Please remove any funding-related text from the manuscript.

5. In the online submission form you indicate that your data is not available for proprietary reasons and have provided a contact point for accessing this data. Please note that your current contact point is a co-author on this manuscript. According to our Data Policy, the contact point must not be an author on the manuscript and must be an institutional contact, ideally not an individual. Please revise your data statement to a non-author institutional point of contact, such as a data access or ethics committee, and send this to us via return email. Please also include contact information for the third party organization, and please include the full citation of where the data can be found.

6. Your ethics statement should only appear in the Methods section of your manuscript. If your ethics statement is written in any section besides the Methods, please move it to the Methods section and delete it from any other section. Please ensure that your ethics statement is included in your manuscript, as the ethics statement entered into the online submission form will not be published alongside your manuscript.

7. We note that Figures 1, 4, 7 and 8 in your submission contain map images which may be copyrighted. All PLOS content is published under the Creative Commons Attribution License (CC BY 4.0), which means that the manuscript, images, and Supporting Information files will be freely available online, and any third party is permitted to access, download, copy, distribute, and use these materials in any way, even commercially, with proper attribution. For these reasons, we cannot publish previously copyrighted maps or satellite images created using proprietary data, such as Google software (Google Maps, Street View, and Earth). For more information, see our copyright guidelines: http://journals.plos.org/plosone/s/licenses-and-copyright.

We require you to either (1) present written permission from the copyright holder to publish these figures specifically under the CC BY 4.0 license, or (2) remove the figures from your submission:

1. You may seek permission from the original copyright holder of Figures 1, 4, 7 and 8 to publish the content specifically under the CC BY 4.0 license.

We recommend that you contact the original copyright holder with the Content Permission Form (http://journals.plos.org/plosone/s/file?id=7c09/content-permission-form.pdf) and the following text:

“I request permission for the open-access journal PLOS ONE to publish XXX under the Creative Commons Attribution License (CCAL) CC BY 4.0 (http://creativecommons.org/licenses/by/4.0/). Please be aware that this license allows unrestricted use and distribution, even commercially, by third parties. Please reply and provide explicit written permission to publish XXX under a CC BY license and complete the attached form.”

Please upload the completed Content Permission Form or other proof of granted permissions as an "Other" file with your submission.

In the figure caption of the copyrighted figure, please include the following text: “Reprinted from [ref] under a CC BY license, with permission from [name of publisher], original copyright [original copyright year].”

2. If you are unable to obtain permission from the original copyright holder to publish these figures under the CC BY 4.0 license or if the copyright holder’s requirements are incompatible with the CC BY 4.0 license, please either i) remove the figure or ii) supply a replacement figure that complies with the CC BY 4.0 license. Please check copyright information on all replacement figures and update the figure caption with source information. If applicable, please specify in the figure caption text when a figure is similar but not identical to the original image and is therefore for illustrative purposes only.

The following resources for replacing copyrighted map figures may be helpful:

USGS National Map Viewer (public domain): http://viewer.nationalmap.gov/viewer/

The Gateway to Astronaut Photography of Earth (public domain): http://eol.jsc.nasa.gov/sseop/clickmap/

Maps at the CIA (public domain): https://www.cia.gov/library/publications/the-world-factbook/index.html and https://www.cia.gov/library/publications/cia-maps-publications/index.html

NASA Earth Observatory (public domain): http://earthobservatory.nasa.gov/

Landsat: http://landsat.visibleearth.nasa.gov/

USGS EROS (Earth Resources Observatory and Science (EROS) Center) (public domain): http://eros.usgs.gov/#

Natural Earth (public domain): http://www.naturalearthdata.com/

8. Please upload a new copy of Figures 1, 4, 5, 6, 7 and 8 as the detail is not clear. Please follow the link for more information:  https://journals.plos.org/plosone/s/figures

9. If the reviewer comments include a recommendation to cite specific previously published works, please review and evaluate these publications to determine whether they are relevant and should be cited. There is no requirement to cite these works unless the editor has indicated otherwise.

**Additional Editor Comments:**

Editorial Recommendation: Major Revision Required

The manuscript addresses an important and underexplored public health issue, examining spatial disparities in esophageal cancer in Ethiopia. Both reviewers agree that the topic is relevant and potentially impactful for policy and targeted interventions. However, significant concerns remain across multiple sections of the manuscript.

The revisions should focus on:

• Title and Abstract: Ensure consistency, correct statistical reporting, and highlight key quantitative findings without exaggeration.

• Introduction: Condense background material, strengthen the link between global context and local relevance, and clearly articulate the research gap and objectives.

• Methods: Improve transparency in study design, sampling, and spatial analysis procedures. Justify methodological choices, acknowledge limitations such as hospital-based data and selection bias, and clarify assumptions.

• Results: Correct inconsistencies, report-missing data explicitly, ensure alignment with the abstract, and interpret findings cautiously with appropriate confidence intervals and uncertainty measures.

• Discussion: Expand and strengthen by comparing findings with regional and international literature, exploring plausible risk factors, and situating results within broader public health implications.

• Ethics and Data Availability: Clarify consent and confidentiality procedures, and revise the data availability statement to comply with journal policy.

• Presentation: Address typographical errors, improve grammar and phrasing, and standardize tables, references, and figures (including maps).

Please address comments and suggestions provided by both reviewers.

[Note: HTML markup is below. Please do not edit.]

Reviewers' comments:

Reviewer's Responses to Questions

**Comments to the Author**

1. Is the manuscript technically sound, and do the data support the conclusions?

Reviewer #1: Yes

Reviewer #2: Yes

**********

2. Has the statistical analysis been performed appropriately and rigorously?

Reviewer #1: Yes

Reviewer #2: Yes

**********

3. Have the authors made all data underlying the findings in their manuscript fully available?

The PLOS Data policy

Reviewer #1: Yes

Reviewer #2: Yes

**********

4. Is the manuscript presented in an intelligible fashion and written in standard English?

Reviewer #1: Yes

Reviewer #2: No

**********

Reviewer #1: 1. Title

Line 1

• The title is clear but could be improved by indicating the analytical methods (e.g., spatial scan statistics, Moran’s I).

• Consider specifying study period if applicable.

• Suggested revision example:

“Spatial Clustering of Esophageal Cancer in Central Oromia, Ethiopia: A Spatial Autocorrelation and Scan Statistics Analysis.”

2. Abstract

Background

Line 6–9

• The sentence structure is unclear and grammatically inconsistent.

• “GLOBOCAN report showed” should be written as “According to the GLOBOCAN 2022 report”.

• The statement “with the two zones having the highest prevalence (68.3%)” needs a clear citation or explanation of the calculation method.

• “Undertaking thorough study” should be “a thorough study”.

Reviewer concern:

• The background mixes global statistics and regional statistics without clear transition.

Objective

Line 12

• The objective is clear but too broad.

• Consider specifying methodological approach.

Example improvement:

“To assess spatial clustering and identify geographic hotspots of esophageal cancer in Central Oromia using spatial autocorrelation and spatial scan statistics.”

Methods

Line 15–21

Issues:

1. Study design

o “The cross-sectional study design was employed” should specify retrospective record review.

2. Sample size

o “367 patients collected from records” should be clarified:

� What years were included?

� Were cases confirmed histologically?

3. Hospital names

o Ensure correct spelling:

o “Tikur Anmbassa” → Tikur Anbessa

4. Geographic variables

o You mention kebele, woreda, and zone, but it is unclear:

� Which level was used in the analysis?

5. Spatial data source

o The Ethiopian Space Science and Geospatial Institute should be cited properly:

o Ethiopian Space Science and Geospatial Institute

Statistical methods

The abstract mentions:

• Gini coefficient

• Moran’s I

• Relative risk

• Log likelihood ratio

Reviewer concern:

• The software used (SaTScan, ArcGIS, R) should be mentioned.

Results

Common issues observed in the manuscript style:

• Results must include numerical values with confidence intervals.

• Avoid vague statements like:

o “significant clustering was observed”.

Better example:

Moran’s I indicated significant spatial autocorrelation (I = 0.34, p < 0.001).

Conclusion

Typical reviewer concern:

• Conclusions must not exaggerate causal interpretation.

• Avoid words like:

o “determinants”

o “cause”.

3. Introduction

Line-by-Line Issues

Line ~35

• The introduction lacks global epidemiological context.

You should cite:

• International Agency for Research on Cancer

• World Health Organization

Line ~40

The statement:

“Esophageal cancer is common in Ethiopia.”

This needs:

• prevalence

• incidence

• mortality

Line ~45

The manuscript should reference the African esophageal cancer belt.

Reviewer expectation:

• Discussion of high-risk areas from:

• African Esophageal Cancer Consortium

Line ~50

Research gap is not clearly stated.

You should answer:

• What spatial studies exist?

• What gap remains?

Example gap statement:

“However, little is known about the spatial distribution and clustering of esophageal cancer within Ethiopia.”

4. Methods

Study Design

Line ~70

• Cross-sectional design is mentioned but time period is missing.

• The reviewer must know:

Study period (e.g., 2015–2023)

Study Area

Line ~80

• Central Oromia must be defined:

Include:

• map

• administrative boundaries

Relevant entity:

• Oromia Region

Data Source

Line ~90

Issues:

• Case definition unclear.

• Were cases:

✔ Histologically confirmed

✔ clinically diagnosed

Inclusion Criteria

Missing information:

• age range

• residence duration

• duplicate records

Geocoding

Line ~100

The process of linking kebele addresses to shapefiles must be explained.

Questions:

• Was GPS used?

• Were coordinates approximated?

Spatial Analysis

Line ~120

Moran’s I should include explanation.

Expected formula:

I = spatial autocorrelation index

Reviewer suggestion:

Explain:

• Weight matrix

• Spatial neighbors

Cluster Detection

If SaTScan was used:

Please cite:

• SaTScan

And explain:

• scanning window

• maximum cluster size

• Monte Carlo simulations

Gini Coefficient

Reviewer concern:

Why is the Gini coefficient used for spatial inequality?

Needs justification.

Ethical Approval

Missing critical information:

• Institutional Review Board approval

• Data confidentiality

Expected example:

Ethical approval was obtained from Arsi University Institutional Review Board.

Relevant entity:

• Arsi University

5. Results

Sample Description

Line ~150

The manuscript should present:

• age distribution

• sex distribution

• residence distribution

A table is required.

Spatial Autocorrelation

Reviewer expects:

• Moran’s I value

• p-value

• interpretation

Maps

Spatial analysis requires clear maps:

Expected figures:

1⃣ Study area map

2⃣ Moran’s I cluster map

3⃣ Hotspot map

4⃣ Relative risk map

Cluster Results

For SaTScan clusters provide:

• cluster center

• radius

• relative risk

• p-value

6. Discussion

Line ~210

The discussion must begin with key findings summary.

Example:

This study identified significant spatial clustering of esophageal cancer in Central Oromia.

Literature Comparison

The manuscript currently lacks comparison with African studies.

Suggested comparisons:

• Kenya

• Tanzania

• Malawi

• Ethiopia previous studies

Possible Explanations

Reviewer expects discussion of:

• diet

• hot beverage consumption

• mycotoxin exposure

• tobacco

• alcohol

Public Health Implications

The discussion should include:

• screening programs

• cancer registry strengthening

• environmental risk assessment

7. Limitations

Important limitations missing:

1. hospital-based data

2. referral bias

3. missing exposure variables

4. geocoding inaccuracies

8. Conclusion

Conclusion should:

✔ summarize findings

✔ avoid speculation

✔ suggest policy action

9. References

Reviewer observations:

• Several citations appear outdated.

• Ensure Vancouver format required by PLOS ONE.

Overall Reviewer Recommendation

Major issues remain in:

1⃣ Methods transparency

2⃣ Spatial analysis explanation

3⃣ Statistical reporting

4⃣ Literature context

Recommendation:

Major Revision Required

Reviewer name: Yohannes Fekadu

Reviewer #2: General comment and overview

The manuscript addresses an important public health issue geographical disparities in esophageal cancer in Ethiopia, using spatial analytical techniques. The topic is relevant and potentially impactful for policy and targeted interventions. However, several methodological inconsistencies, reporting gaps, and clarity issues need substantial revision before publication.

2. Title and Abstract

Strengths

�The title is clear and reflects the study design and scope and the abstract includes major sections (background, methods, results, conclusion).

Major Concerns

Abstract inconsistency and statistical errors:

�The confidence interval for low-risk districts is incorrect: RR = 0.55; 95% CI: 1.81–2.10 (Page 2, lines 47–48). The CI contradicts the RR and must be corrected.

�Methods description is too dense and lacks clarity (Page 1–2, lines 31–39).

�The conclusion is vague and does not clearly reflect key quantitative findings.

Recommendations

�Correct all statistical inconsistencies.

�Simplify and structure the methods section in the abstract.

�Include clear quantitative highlights (e.g., strongest cluster, magnitude of inequality).

3. Introduction / Background

Strengths

�Provides global and regional context with relevant statistics.

�Clearly identifies the research gap (lack of spatial modeling in Ethiopia).

Major Issues

�Excessive length and redundancy (Pages 2–4, lines 60–106).

�Some statements lack proper linkage to study objectives.

�Overuse of global statistics without sufficient focus on local relevance.

Recommendations

�Condense global burden discussion.

�Strengthen linkage between background → gap → study objective.

�Clearly justify why Central Oromia is uniquely important.

4. Research Questions and Hypotheses

Strengths

�Clearly stated research questions and null hypotheses (Page 5, lines 131–137).

Issues

�Hypotheses are simplistic and do not fully reflect spatial modeling complexity.

Recommendations

�Consider including alternative hypotheses and clarifying spatial assumptions.

5. Methods

5.1 Study Design and Data Source

Strengths

�Multi-center data source improves coverage.

Concerns

�Cross-sectional design limits causal inference (Page 7, lines 184–188).

�Hospital-based data may introduce selection bias (referral bias not discussed).

Recommendation

�Explicitly acknowledge selection bias and limited representativeness.

5.2 Sampling and Sample Size

Major Issues

�Sample size calculation is unclear and not aligned with spatial analysis (Page 7–8, lines 197–210).

�Inclusion of all 367 cases contradicts initial sample size calculation logic.

�Use of StatsToDo tool is insufficiently justified scientifically.

Recommendations

�Clarify:

�Why Poisson-based sample size is appropriate.

�Why all cases were included despite calculated minimum.

�Consider reframing as complete case inclusion rather than sample-based study.

5.3 Data Collection and Measurement

Strengths

�Use of multiple data sources and trained collectors.

Concerns

�Verification of patient status via phone (Page 8, lines 226–227) raises:

�Ethical concerns (consent unclear)

�Potential recall bias

�High proportion of missing clinical variables (later seen in results).

Recommendations

�Clarify consent procedures for follow-up contact.

�Describe how missing data were handled.

5.4 Spatial Analysis Methods

Strengths

�Appropriate use of: Moran’s I, LISA, Gini coefficient, SaTScan

Major Issues

�Formulas are unnecessarily detailed and disrupt readability (Pages 9–10).

�No justification for: Choice of spatial weight matrix and 50% cluster size threshold (Page 11, line 305)

�Lack of explanation of: Model assumptions (Poisson model in SaTScan)

�Clearly describe model assumptions and diagnostics.

5.5 Data Quality Assurance

Strengths

�Includes training, pretesting, and validation.

Concerns

�“Face validity” and “content validity” lack methodological rigor (Page 12, lines 336–339).

�No reliability testing reported.

Recommendation

�Include inter-rater reliability or consistency checks.

6. Results

6.1 Descriptive Findings

Strengths

�Comprehensive demographic and clinical description.

Major Issues

�Numerous typographical errors:

�“frmales” (Page 13, line 361)

�“Recidence” (Page 14)

�“Wieght loss” (Page 15)

�High proportion of missing data:

�Tumor differentiation (74%)

�Survival status (29%)

Recommendation

�Report missing data explicitly and discuss implications.

6.2 Statistical and Spatial Results

Strengths

�Multi-level spatial analysis (kebele, district, zone).

�Identification of hotspots and cold spots.

Major Issues

�Inconsistent reporting:

�Some results contradict abstract.

�Interpretation of Moran’s I:

�Woreda level non-significant but still discussed as meaningful (Page 18–19).

�Overinterpretation of LISA clusters without uncertainty discussion.

Recommendations

�Ensure consistency between abstract and results.

�Interpret non-significant findings cautiously.

�Include maps with confidence or uncertainty indicators.

7. Discussion (Implicit / Missing Section)

Major Gap

�The discussion section is not fully presented in the provided text but appears underdeveloped.

Expected Improvements

�Compare findings with:

�East African esophageal cancer corridor literature.

�Discuss: Environmental and behavioral risk factors and Health system access issues

8. Ethical Considerations

Strengths

�Ethical approval clearly stated with protocol numbers.

Concerns

Lack of clarity on:

Consent for patient follow-up contact

Data confidentiality procedures beyond storage

9. Data Availability

Major Issue

�Statement contradicts journal policy:

“Available upon request” is not compliant with PLOS ONE policy.

Recommendation

�Deposit data in a public repository or provide justified restriction.

**********

what does this mean?). If published, this will include your full peer review and any attached files.

If you choose “no”, your identity will remain anonymous but your review may still be made public.

**Do you want your identity to be public for this peer review?**  For information about this choice, including consent withdrawal, please see our Privacy Policy

Reviewer #1: **Yes:** Yohannes Fekadu

Reviewer #2: No

**********

[NOTE: If reviewer comments were submitted as an attachment file, they will be attached to this email and accessible via the submission site. Please log into your account, locate the manuscript record, and check for the action link "View Attachments". If this link does not appear, there are no attachment files.]

To ensure your figures meet our technical requirements, please review our figure guidelines: https://journals.plos.org/plosone/s/figures

You may also use PLOS’s free figure tool, NAAS, to help you prepare publication quality figures: https://journals.plos.org/plosone/s/figures#loc-tools-for-figure-preparation.

NAAS will assess whether your figures meet our technical requirements by comparing each figure against our figure specifications.

Attachment Submitted filename: review.docx

Attachment Submitted filename: General comment and overview.docx

# Author comment: Author response to Decision Letter 1

**DOI:** 10.1371/journal.pone.0357042.r002

10 Jun 2026

We are grateful to the reviewers for addressing all sections of our manuscript. We are pleased that our manuscript was reviewed by experts and has been greatly enhanced. We have now revised the methods to improve the transparency of spatial analysis and statistical reporting. Additionally, we have carefully revised the literature section according to the reviewers’ request. In general, we have revised the entire manuscript as requested by the journal's editor and the two reviewers. Our responses to both reviewers are given for each comment one by one. If more clarifications are needed, we are happy to provide them upon request.

Attachment Submitted filename: Response to Reviewers.docx

# Peer review: Decision Letter 1

**DOI:** 10.1371/journal.pone.0357042.r003

**Authors:** Takele Gezahegn Demie

11 Aug 2026

Spatial clustering of esophageal cancer in central Oromia, Ethiopia: A spatial autocorrelation and scan statistics analysis

PONE-D-26-03029R1

Dear Dr. BEYEN,

We’re pleased to inform you that your manuscript has been judged scientifically suitable for publication and will be formally accepted for publication once it meets all outstanding technical requirements.

Within one week, you’ll receive an e-mail detailing the required amendments. When these have been addressed, you’ll receive a formal acceptance letter and your manuscript will be scheduled for publication.

An invoice will be generated when your article is formally accepted. Please note, if your institution has a publishing partnership with PLOS and your article meets the relevant criteria, all or part of your publication costs will be covered. Please make sure your user information is up-to-date by logging into Editorial Manager at Editorial Manager® and clicking the ‘Update My Information' link at the top of the page. For questions related to billing, please contact billing support.

If your institution or institutions have a press office, please notify them about your upcoming paper to help maximize its impact. If they’ll be preparing press materials, please inform our press team as soon as possible -- no later than 48 hours after receiving the formal acceptance. Your manuscript will remain under strict press embargo until 2 pm Eastern Time on the date of publication. For more information, please contact onepress@plos.org.

Kind regards,

Takele Gezahegn Demie, MPH

Academic Editor

PLOS One

Additional Editor Comments (optional):

Congratulations on successfully revising your manuscript. The reviewers’ comments have been thoroughly addressed, and the manuscript is now acceptable for publication. Thank you for your efforts and commitment to improving the work.

Reviewers' comments:

Reviewer's Responses to Questions

**Comments to the Author**

Reviewer #1: All comments have been addressed

Reviewer #2: All comments have been addressed

**********

2. Is the manuscript technically sound, and do the data support the conclusions?

Reviewer #1: Yes

Reviewer #2: Yes

**********

3. Has the statistical analysis been performed appropriately and rigorously?

Reviewer #1: Yes

Reviewer #2: Yes

**********

4. Have the authors made all data underlying the findings in their manuscript fully available?

The PLOS Data policy

Reviewer #1: Yes

Reviewer #2: Yes

**********

5. Is the manuscript presented in an intelligible fashion and written in standard English?

Reviewer #1: Yes

Reviewer #2: Yes

**********

Reviewer #1: (No Response)

Reviewer #2: Thank you for addressing all the comments. All the comments were addressed and incorporated and almost all parts are done well.

**********

what does this mean?). If published, this will include your full peer review and any attached files.

If you choose “no”, your identity will remain anonymous but your review may still be made public.

**Do you want your identity to be public for this peer review?**  For information about this choice, including consent withdrawal, please see our Privacy Policy

Reviewer #1: **Yes:** Yohannes Fekadu

Reviewer #2: No

**********

# Editor report: Acceptance letter

**DOI:** 10.1371/journal.pone.0357042.r004

**Authors:** Takele Gezahegn Demie

PONE-D-26-03029R1

PLOS One

Dear Dr. Beyen,

I'm pleased to inform you that your manuscript has been deemed suitable for publication in PLOS One. Congratulations! Your manuscript is now being handed over to our production team.

At this stage, our production department will prepare your paper for publication. This includes ensuring the following:

* All references, tables, and figures are properly cited

* All relevant supporting information is included in the manuscript submission,

* There are no issues that prevent the paper from being properly typeset

You will receive further instructions from the production team, including instructions on how to review your proof when it is ready. Please keep in mind that we are working through a large volume of accepted articles, so please give us a few days to review your paper and let you know the next and final steps.

Lastly, if your institution or institutions have a press office, please let them know about your upcoming paper now to help maximize its impact. If they'll be preparing press materials, please inform our press team within the next 48 hours. Your manuscript will remain under strict press embargo until 2 pm Eastern Time on the date of publication. For more information, please contact onepress@plos.org.

You will receive an invoice from PLOS for your publication fee after your manuscript has reached the completed accept phase. If you receive an email requesting payment before acceptance or for any other service, this may be a phishing scheme. Learn how to identify phishing emails and protect your accounts at https://explore.plos.org/phishing.

If we can help with anything else, please email us at customercare@plos.org.

Thank you for submitting your work to PLOS One and supporting open access.

Kind regards,

PLOS One Editorial Office Staff

on behalf of

Mr. Takele Gezahegn Demie

Academic Editor

PLOS One
